# Benchmarking Water Models in Molecular Dynamics of Protein–Glycosaminoglycan Complexes

**DOI:** 10.1021/acs.jcim.4c00030

**Published:** 2024-02-27

**Authors:** Sebastian Anila, Sergey A. Samsonov

**Affiliations:** Faculty of Chemistry, University of Gdańsk, ul. Wita Stwosza 63, 80-308 Gdańsk, Poland

## Abstract

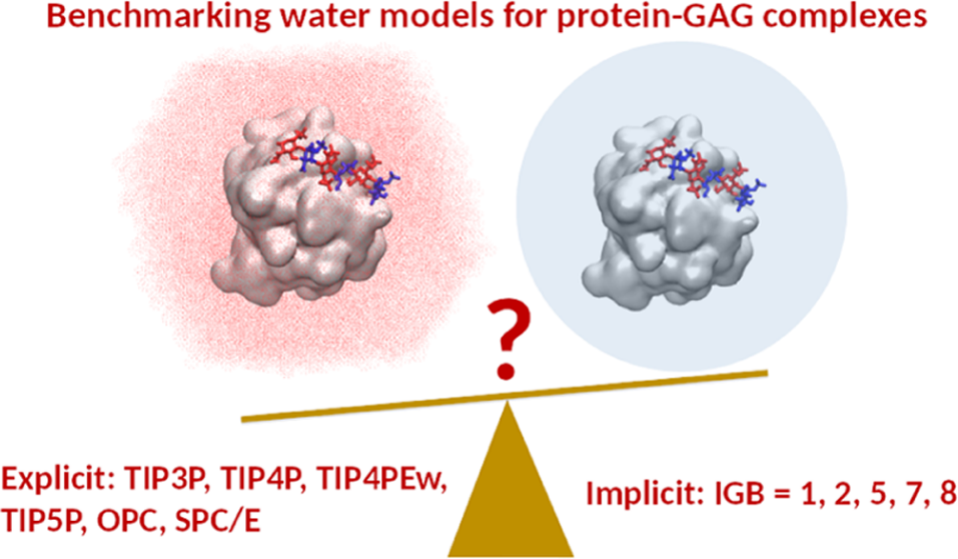

Glycosaminoglycans (GAGs) made of repeating disaccharide units intricately engage with proteins, playing a crucial role in the spatial organization of the extracellular matrix (ECM) and the transduction of biological signals in cells to modulate a number of biochemical processes. Exploring protein–GAG interactions reveals several challenges for their analysis, namely, the highly charged and periodic nature of GAGs, their multipose binding, and the abundance of the interfacial water molecules in the protein–GAG complexes. Most of the studies on protein–GAG interactions are conducted using the TIP3P water model, and there are no data on the effect of various water models on the results obtained in molecular dynamics (MD) simulations of protein–GAG complexes. Hence, it is essential to perform a systematic analysis of different water models in MD simulations for these systems. In this work, we aim to evaluate the properties of the protein–GAG complexes in MD simulations using different explicit: TIP3P, SPC/E, TIP4P, TIP4PEw, OPC, and TIP5P and implicit: IGB = 1, 2, 5, 7, and 8 water models to find out which of them are best suited to study the dynamics of protein–GAG complexes. The FF14SB and GLYCAM06 force fields were used for the proteins and GAGs, respectively. The interactions of several GAG types, such as heparin, chondroitin sulfate, and hyaluronic acid with basic fibroblast growth factor, cathepsin K, and CD44 receptor, respectively, are investigated. The observed variations in different descriptors used to study the binding in these complexes emphasize the relevance of the choice of water models for the MD simulation of these complexes.

## Introduction

Glycosaminoglycans (GAGs) stand out among diverse functional polymers within human cells. These long linear periodic negatively charged polydisperse polysaccharides complexly engage with proteins, playing a crucial role in the processes of the extracellular matrix (ECM).^1^ Natural as well as chemically modified GAG have gained significant attention for their potential in biomaterial design.^2,3^ Their application holds promise for adopting biospecific cell behavior, particularly in the realms of skin and bone tissue regeneration.^4−6^ GAGs are made of repeating disaccharide units, each of which consists of a hexuronic acid (or galactose in keratan sulfate) and a hexosamine (*N*-acetylglycosamide, GlcNAc or *N*-acetylgalactososamide GalNAc) connected with 1–4 or 1–3 glycosidic linkages, while hydroxyl groups of hexose and hexosamine at various positions can be sulfated. Based on the core disaccharide structures, GAGs can be classified as heparin (HP), heparan sulfate (HS), chondroitin sulfate (CS), dermatan sulfate (DS), hyaluronic acid (HA), and keratan sulfate (KS).^7^ The disaccharide units of HP and HS are both structurally composed of alternating 4-linked uronic acid (GlcA/IdoA) and 4-linked α-glucosamine (GlcN) units.^8^ CS disaccharide units are composed of alternating 4-linked β-d-GlcA and 3-linked β-d-galactosamine (GalNAc) units. Among various CS subtypes, owing to structural variations: CS-A is mostly 4-sulfated at the GalNAc units, while CS-C is predominantly 6-sulfated, and CS-B, widely known as DS, has α-l-IdoA units rather than β-d-GlcA. The IdoA units in DS may bear 2-sulfation, while the GalNAc units are mostly 4-sulfated.^9^ KS disaccharide units are composed of alternating 3-linked β-d-Gal and 4-linked β-d-GlcNAc units.^10^ Unlike other GAGs, HA lacks sulfate groups and is not covalently bound to a protein. It is composed of repeating disaccharide units of alternating 4-linked β-d-GlcA and 3-linked β-d-GlcNAc.^11^

Based on the sulfation pattern and monosaccharide composition, GAG disaccharide units can display 408 variants.^12^ The variations in the monosaccharide composition and the sulfation patterns of the GAGs may alter their binding and functional properties as well as conformational characteristics.^13^ GAGs are mainly located in the extracellular matrix. Although they were once thought of as a kind of inert glue around cells, recent research has shown that GAGs play a vital role in building biological systems and the transduction of biological signals in cell proliferation, regeneration, lipid metabolism, angiogenesis, and metastasis.^14,15^ All these processes are mediated through their direct interactions with diverse protein targets such as collagens, chemokines, cytokines, growth factors, antithrombin, and cell adhesion molecules, which makes them essential players in cell biology.^16−20^

The participation of GAGs in physiological, pathological, or therapeutic functions results principally from their unique physicochemical and structural features, including high negative charge, high viscosity, lubricative properties, periodicity, unbranched polysaccharide structures, low compressibility, and the ability to attract and absorb enormous amounts of water.^21^ Analysis of the interaction of GAG with proteins is required for understanding various physiological and pathological mechanisms and is a huge stimulus for drug development. Most protein–GAG interactions are driven by electrostatics and are nonspecific,^22,23^ whereas some of them, in contrast, can be highly specific^24^ or selective.^25^ Computational approaches rapidly developing for carbohydrates in recent decades including the emergence of new force fields and scoring functions are useful for a detailed analysis of the structure–function relationships underlying the mechanisms of protein–GAG interactions and for gaining a better understanding of the molecular bases of carbohydrate recognition.^26−29^ However, there are still many challenges for computational studies of protein–GAG interactions. GAGs are highly charged molecules; therefore, electrostatics should be treated appropriately.^30^ Also, the interfacial water molecules are crucial in defining the GAG binding pose, and hence, the solvent-mediated interactions should be accurately taken into account for protein–GAG interaction analysis due to their abundance.^31,32^ Precisely predicting the strength and nature of protein–GAG interactions remains intangible also due to the need for comprehensive analyses of the conformational space of the flexible positively charged amino acid residues recognizing negatively charged GAGs. The presence of multiple binding sites in a single protein structure for either the same or different GAGs further complicates this task.^33^ The dynamic nature of the protein–GAG interactions with different ranges of specificity underlines the complexity inherent in interpreting the mechanisms in which they are involved, emphasizing the necessity for sophisticated approaches in the study of these molecular interactions.

The solvent effect in the molecular docking and molecular dynamics (MD) simulations can be considered by using either explicit or implicit water models. The explicit solvent model represents the solvent with individual atomistic solvent molecules surrounding the solute. In contrast, an implicit solvent model mimics the presence of a solvent in an average manner as a continuous medium surrounding the solute. In Amber, generalized Born (GB) is the most common implicit solvent model. This model is based on an approximation to the exact (linearized) Poisson–Boltzmann equation.^34,35^ Recent studies have shown that GB models do not reproduce well some basic molecular properties like the secondary structures of *de novo* designed peptides.^36^ Most of the MD-related studies on GAGs are conducted using the TIP3P water model, as it is widely accepted in the GAG field and proven to be working well in the protein–GAG systems, as well as in MD studies of other biomolecular systems in general.^37−39^ The basic reason for the wide use of TIP3P and other three-site water models is their low computational cost compared to four- and five-site solvent models. Although some studies suggest the use of a more advanced water model than three-site TIP3P, e.g., TIP4P or TIP5P, there are no data on the use of different water models for protein–GAG complexes, and thus, there is no evidence of which other model should be more appropriate for these systems.^40,41^ A comparison of the HP properties in the MD simulations with SPC and SPC/E water models was reported, stating the superiority of SPC/E over the SPC water model.^42^ The comparison of various explicit water models for modeling CS by Neamtu et al. suggested TIP4P and TIP4PEw water models as the most appropriate ones.^43^ Recently, Marcisz and Samsonov reported a detailed evaluation of the properties of the HP in different explicit and implicit water models.^44^ Their study has shown that TIP5P and OPC water models allowed the best agreement with the experiment for both local and global structural features of HP in the MD simulations.

Since explicit water models can improve the performance of molecular docking and MD simulations,^31,45^ currently, implicit water models are used less frequently, especially due to easier access to high-performance computing facilities than before. With the emerging computing facilities, the computational costs can be surpassed to greater extents, and hence, even five-point or more computationally expensive water models can be used in the analysis of the protein–GAG complexes. However, owing to their low computational cost, implicit solvent models are still utilized when computational resources and time are limited or the size of the studied system is particularly large. Thus, it is essential to obtain the data from a systematic analysis of the effect of different water models on the MD of protein–GAG complexes.

In this work, we aim to study the properties of the protein–GAG complexes with MD simulations using different explicit and implicit water models to find out which of them are best suited for these complexes. In the present work, all-atom MD simulations are conducted for 10 μs to study the dynamics of the protein–GAG complexes, complemented by free energy analysis in different water models. The free energy analysis of the protein–GAG interactions is important to understand the nature of the interactions and the stability of the binding pose, especially in the case of complexes with multiple binding poses. Here, the binding interactions of the GAGs: HP, CS, and HA with basic fibroblast growth factor, cathepsin K, and CD44 receptors, respectively, are investigated using six different explicit and five implicit water models. The selection of protein–GAG complexes was deliberate, considering their diverse ranges of binding affinity. The basic fibroblast factor–HP complex exhibits a very strong binding. In contrast, the cathepsin K–CS complex involves moderate binding affinity, while the CD44–HA complex formation is driven by weak interactions. These three complexes therefore provide a spectrum of binding strengths. The cathepsin K–CS complex has two binding poses, as observed experimentally in crystal structures (PDB IDs: 4N8W and 3C9E). This adds an intriguing dimension, prompting an investigation of whether two binding poses can be distinguished in the MD simulations using various water models. The exploration of such changes can offer valuable insights into the dynamic behavior of these systems in MD simulations.

## Materials and Methods

The initial structure of the protein–GAG complexes used in this study was obtained from the Protein Data Bank (PDB IDs: 1BFC, 2.20 Å; 4N8W, 2.02 Å; 3C9E, 1.80 Å; 2JCQ, 1.25 Å).

### Water Models

All of the parameters for the water models used in this work are taken from Amber20,^46^ and recommended mbondii are used for each particular model.^47^ Explicit: TIP3P,^48,49^ SPC/E,^50^ TIP4P,^48^ TIP4PEw,^51^ OPC,^41^ and TIP5P,^52^ and implicit IGB = 1,^53^ 2,^54^ 5,^55^ 7,^56^ and 8^35^ water models are used.

### MD Simulations

All-atom MD simulations of the protein–GAG complexes are performed in the AMBER20 package. FF14SB and GLYCAM06 force field parameters were used for the protein and GAGs, respectively. In the case of explicit solvent models, a truncated octahedron water box of the respective water model with a 12 Å distance from the solute to the box’s border is used to solvate complexes. Na^+^/Cl^–^ counterions are used to neutralize the charge of the system. No “saltcon” option is used in the implicit solvent simulations. Two steps of energy minimization are performed: first 500 steepest descents and then 1000 conjugate gradient cycles with harmonic restraints of 100 kcal/mol/Å^2^ on the solute, followed by 3000 steepest descent and 3000 conjugate gradient cycles without restraints for the explicit solvent simulations, while only the second minimization step is performed for the implicit solvent simulations. Then, the system is heated to 300 K for 10 ps with harmonic restraints of 100 kcal/mol/Å^2^ on the solute and equilibrated for 100 ps at 300 K and 10^5^ Pa in an isothermal isobaric ensemble (NTP) for the explicit solvent simulation. Afterward, MD simulations are carried out in the same NTP ensemble for 10 μs. A 2 fs time integration step and an 8 Å cutoff for nonbonded interactions are used. The SHAKE algorithm for all of the covalent bonds containing hydrogen atoms^57^ and the Particle Mesh Ewald method to treat electrostatics were used.^58^ The trajectories are analyzed by the cpptraj module of AmberTools.^59^ In particular, the *native contacts* command with default parameters is used for the analysis of the contacts between the protein and GAG molecules established during the simulation and for their comparison with the ones in experimental structures.

### Binding Free Energy Calculations

Binding free energy calculations are performed for entire MD trajectories using molecular mechanics generalized Born surface area (MM/GBSA) and a model with the surface area and Born radii as default parameters as implemented in IGB = 2 model^47^ in AMBER20.

### GAG’s Binding Poses Accuracy Evaluation

For the evaluation of the binding pose accuracy in this work, root mean square deviation (RMSD) and root mean square atom type distance (RMSatD) values are used. Both RMSD and RMSatD are measures of structural similarity between different conformations of the biomolecules. RMSatD accounts for the equivalence of the atoms of the same atomic type and, therefore, is more appropriate for the periodic GAG molecules than RMSD.^32^

### Data Analysis and Visualization

The data are analyzed, and the figures are prepared with the R-package.^60^ Structures and trajectories are analyzed with VMD.^61^

## Results

### Basic Fibroblast Factor–HP Complex

**Figure 1:**
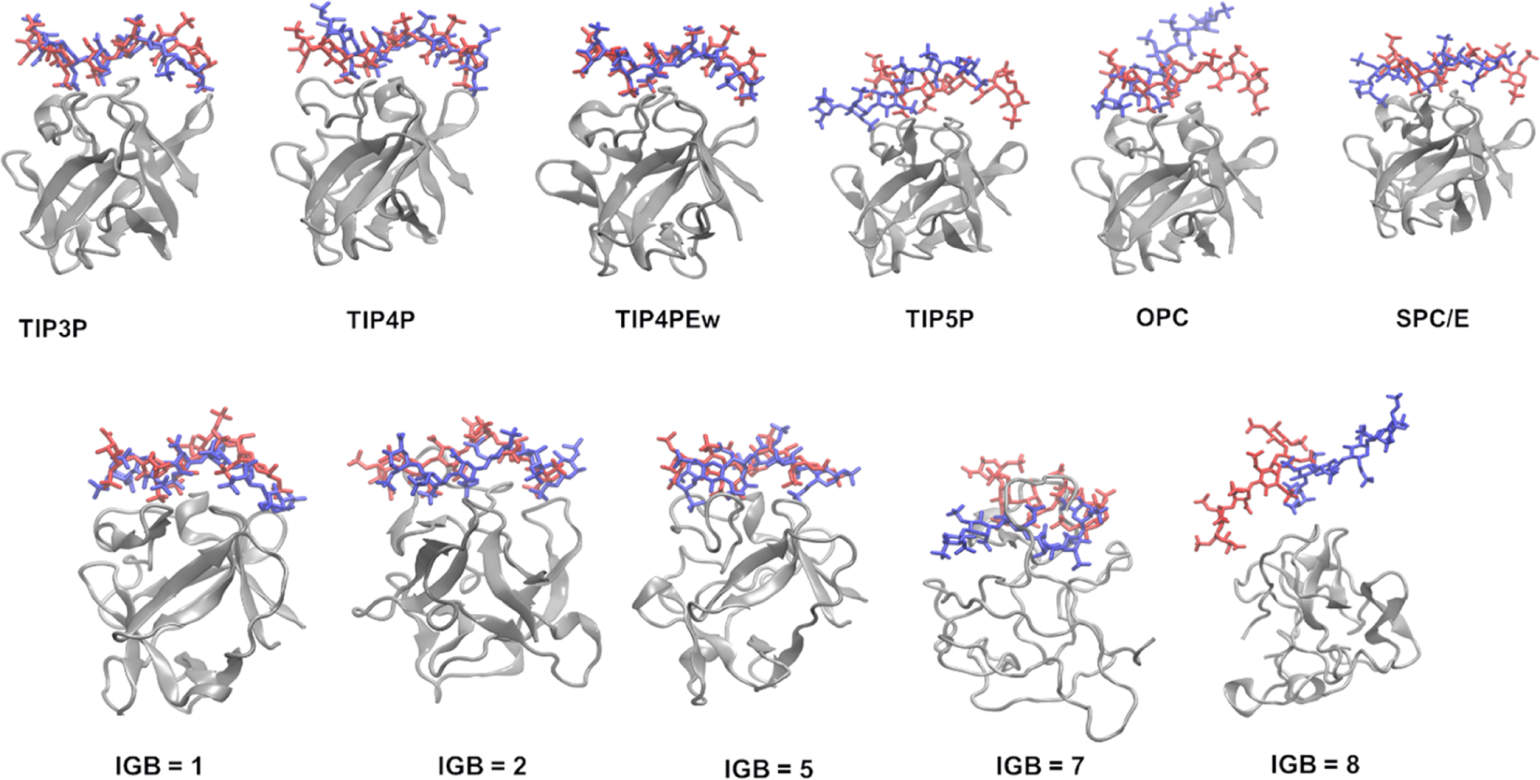
HP starting (in red, licorice) and final (in blue, licorice) poses in the complex with the basic fibroblast factor (in silver, new cartoon).

The structural analysis reveals a similarity in the structures of the basic fibroblast factor–HP complex at the simulation’s beginning and end when utilizing water models such as TIP3P, TIP4P, TIP4PEw, and SPC/E (Figure 1). In contrast, a notable deviation in the ligand binding at the beginning and end of the simulation is observed specifically for the simulations using TIP5P and the OPC water models. This disparity underlines the sensitivity of the simulation outcome to the choice of water models. The simulations involving the basic fibroblast factor–HP complex exhibited more pronounced alterations when implicit water models were used. In contrast to the use of explicit water models, where the protein structure remained stable throughout the simulation, the use of implicit water models induced notable changes in the protein’s conformation, especially in the simulation using the IGB = 7 water model.

### Analysis of the Complex Stability

**Figure 2:**
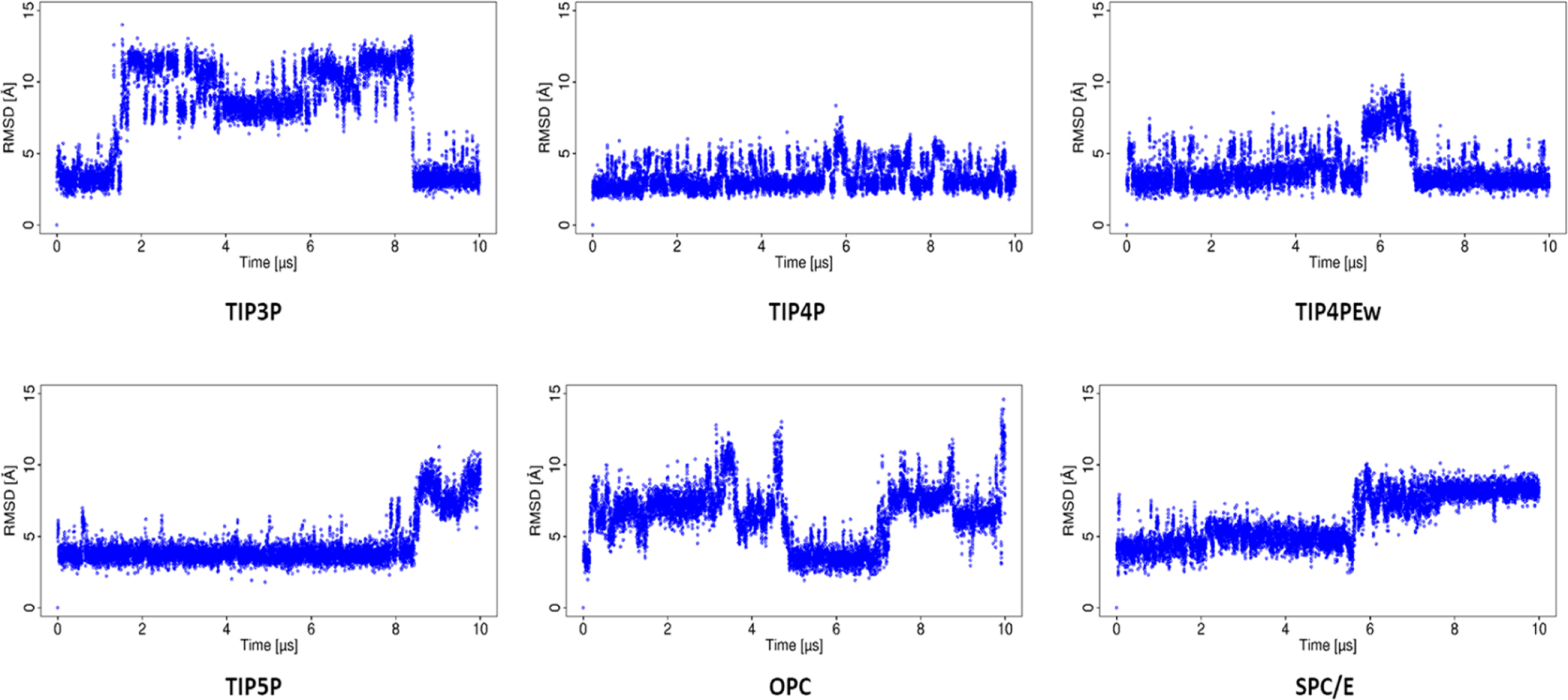
RMSD of the ligand obtained for the basic fibroblast factor–HP complex in MD simulations with explicit water models.

The RMSD analysis provides a comprehensive assessment of the changes in the protein and ligand structures with respect to the starting structure in the MD simulations with different water models (Figure 2). In the case of TIP3P, after 2 μs of the simulation, the ligand shows remarkably high RMSD, indicating a notable change in the binding pose of the HP, and after 8 μs, the RMSD drops to lower magnitudes, which indicates the arrangement of the ligand with the protein in a very similar way to that at the beginning of the simulation. For the simulations using TIP4P and TIP4PEw water models, the RMSD of the ligand is in a similar range of values, indicating that the arrangement of the ligand with respect to the protein is not significantly altered. In the case of simulations using the TIP5P water model, very low RMSD is observed for the ligand up to 8 μs and then shows a sudden increase. With the OPC water model, the MD simulation shows more frequent fluctuations in terms of RMSD, while in the simulation with the SPC/E water model, the ligand orientation gradually changes from the beginning of the simulation. RMSD of the protein was around 2 Å using explicit water models, which shows that the protein is stable in the entire simulation (Figure S1).

**Figure 3:**
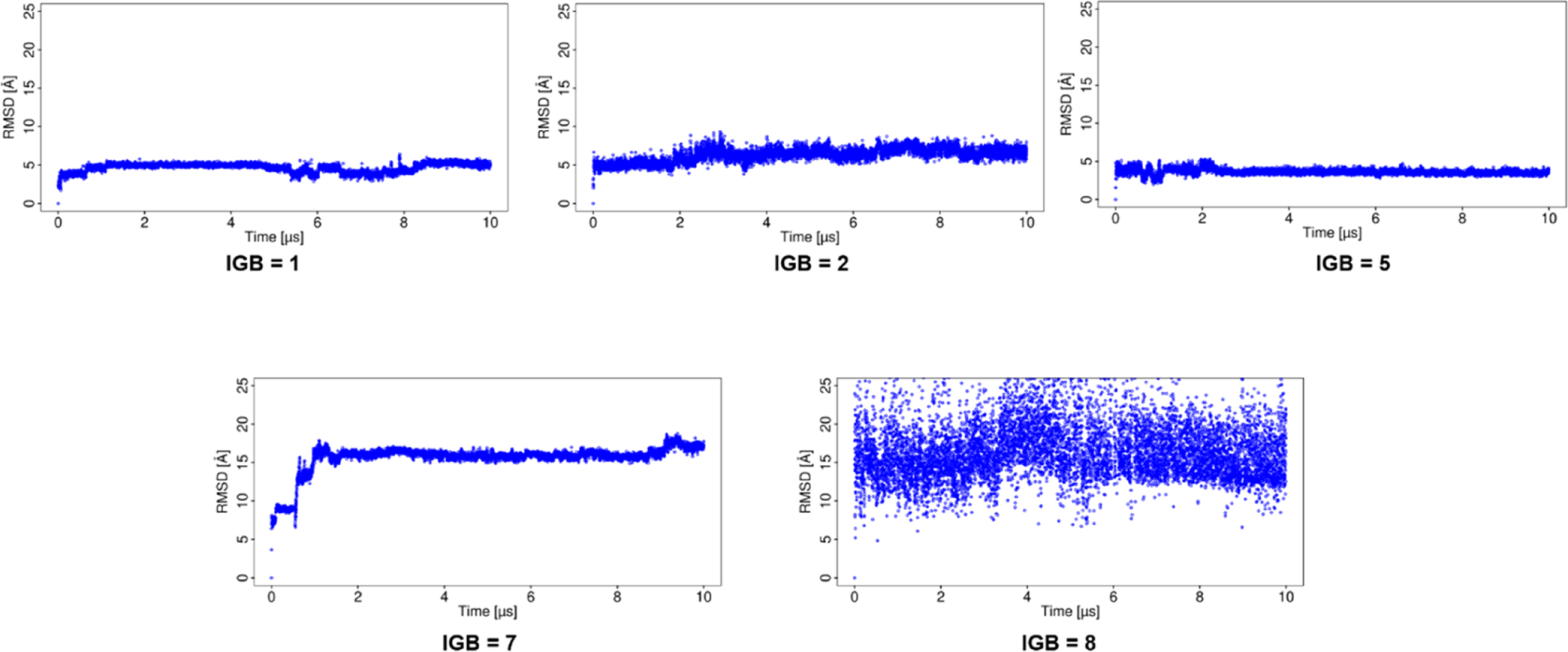
RMSD of the ligand for the basic fibroblast factor–HP complex in MD simulations with implicit water models.

The RMSD of the ligand from the MD simulation using implicit water models (IGB = 1, 2, and 5 models; Figure 3) is lower compared to that of the simulations using IGB = 7 and 8, suggesting that the use of the later ones results in significant changes in the binding pose. The RMSD of the protein from the MD simulation using implicit water models showed that the protein structure is significantly altered in most of them (Figure S2). In the case of the MD simulation with IGB = 1, both the ligand and the protein showed low values of RMSD (4.7 ± 0.6 and 2.6 ± 0.4 Å, for the ligand and protein, respectively), indicating the complex stability. For the MD simulation with IGB = 2 and 5, both the ligand and protein deviate more from their starting structures than in the simulation with IGB = 1. The simulations with IGB = 7 and 8 showed a maximum RMSD for both the ligand and protein. The RMSDs for the ligand and protein are provided in Table S1.

### Binding Free Energy Analysis

**Figure 4:**
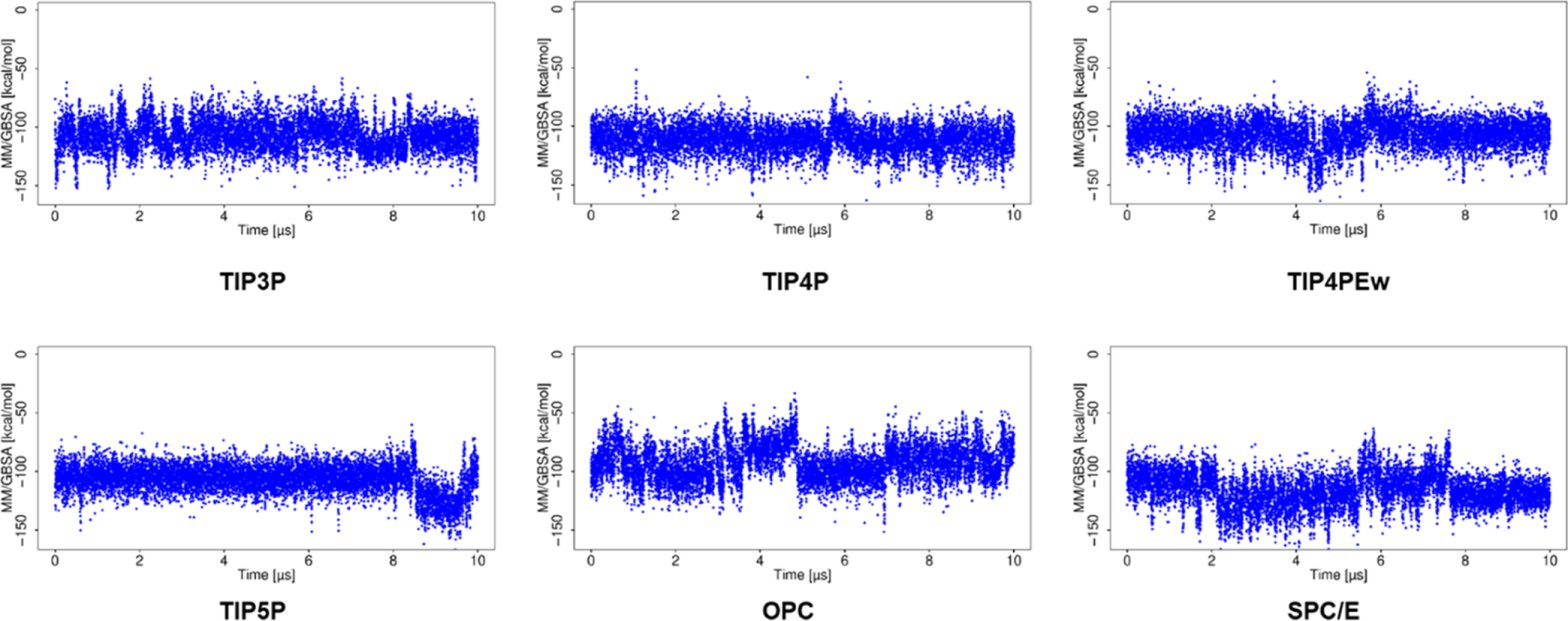
Δ*G* obtained using MM/GBSA analysis for the basic fibroblast factor–HP complex in MD simulations with explicit water models.

**Figure 5:**
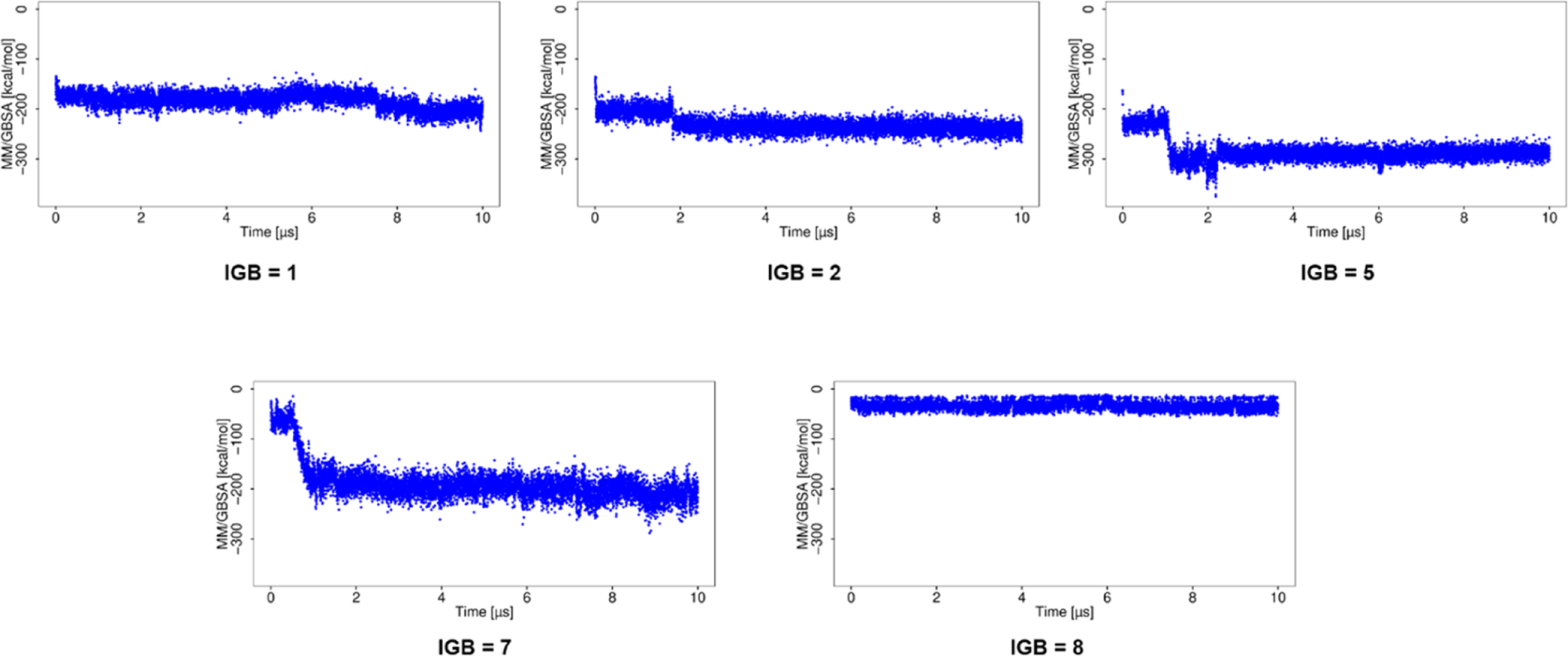
Δ*G* for the basic fibroblast factor–HP complex in MD simulations with implicit water models.

To estimate the effect of these structural variations on the stability of the complex, the binding free energy (Δ*G*) of the complex in the simulations with various water models has been calculated using MM/GBSA (Figures 4 and 5). In the case of the MD simulation using TIP3P, irrespective of the changes in RMSD, Δ*G* showed a similar range of values during the entire 10 μs simulation. For MD simulations using TIP4P and TIP4Pew, Δ*G* becomes less favorable with large RMSD observed for the ligand. Even the small variations in RMSD are also reflected in the MM/GBSA data with a corresponding change in Δ*G*. For simulations using TIP5P and SPC/E, higher deviations from the initial structure correspond to the more favorable free energy of binding. For the simulation with the OPC water model, Δ*G* is of higher variance, which agrees with the more fluctuating RMSD observed for the ligand.

The MD simulations using implicit water models showed a more favorable Δ*G* (Figure 5) than that observed with explicit water models. For the simulation using IGB = 1, Δ*G* becomes more favorable with the increase of RMSD. For the simulation using IGB = 2, 5, and 7, high RMSD corresponded to more favorable Δ*G*, suggesting complex stabilization. The MD simulation with water model IGB = 8 yields high RMSD for the ligand and less favorable Δ*G*, suggesting destabilization of the complex.

### Analysis of Contacts and H-Bonds

**Figure 6:**
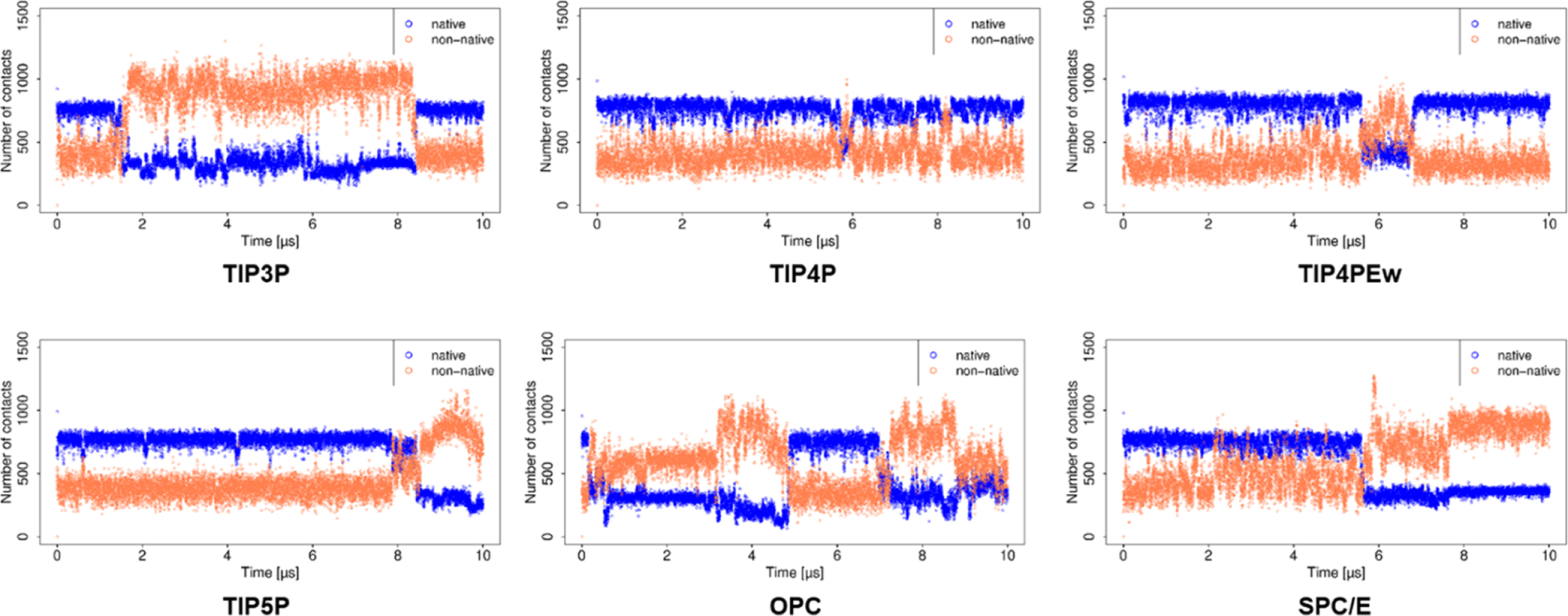
Number of contacts obtained for the basic fibroblast factor–HP complex in MD simulations with explicit water models.

**Figure 7:**
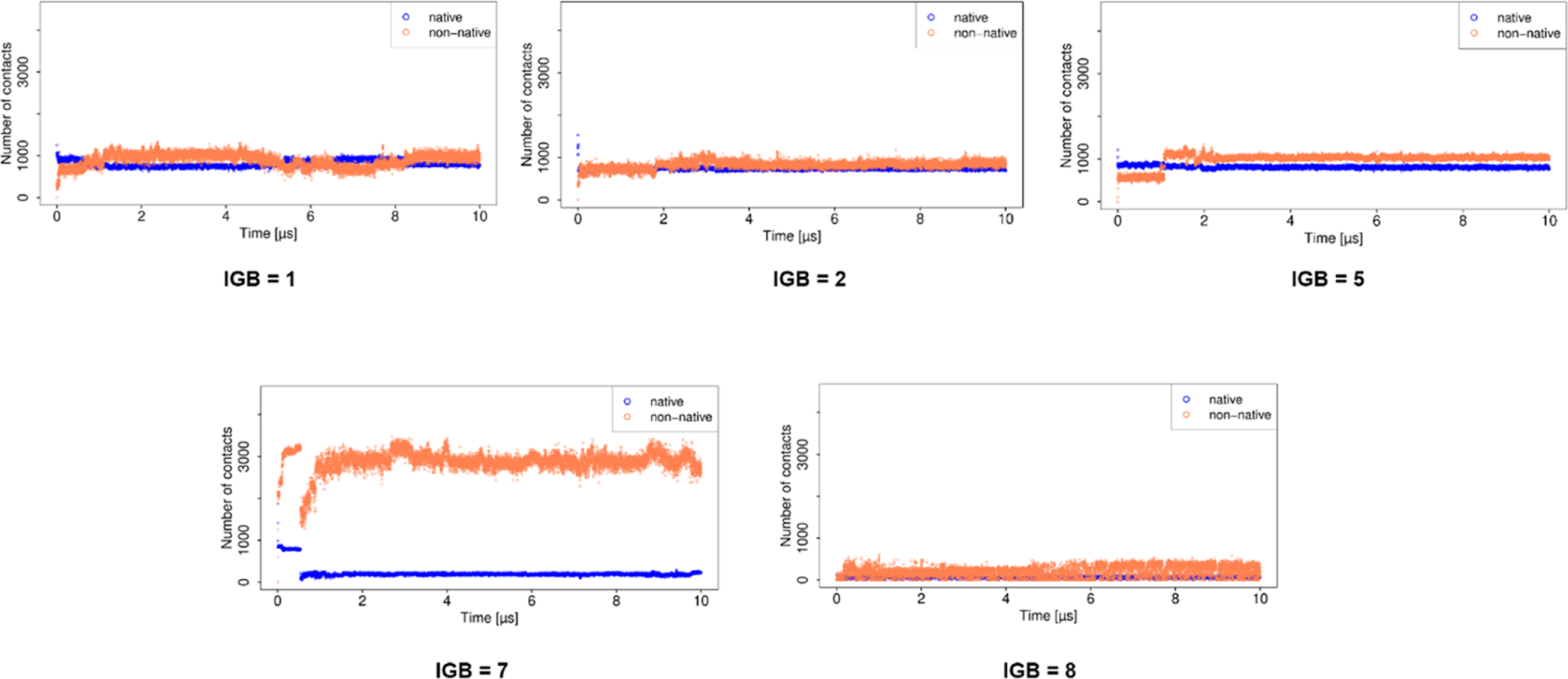
Number of contacts obtained for the basic fibroblast factor–HP complex in MD simulations with implicit water models.

Analysis of contacts (Figures 6 and 7) showed a trend similar to that observed from the RMSD and MM/GBSA analyses for the simulations using explicit water models. Especially the abrupt changes in these parameters observed with TIP3P and TIP5P water models are reflected in the change in the number of contacts. Similarly, the H-bond analysis also shows a lower number of H-bonds with increased RMSD values. The stabilization effects for the complex with the TIP5P model with increased RMSD can therefore be explained by the increased number of H-bonds (Figure S3).

In the case of MD simulations using implicit water models, the stabilization of the complex can be explained as a result of the increased number of contacts and H-bonds (Figure S4). After the initial drop in the number of native contacts, for the simulations using IGB = 1, 2, and 5, the number of native contacts remains steady, whereas the number of non-native contacts increases gradually. In the case of the simulation using IGB = 7, although the number of native contacts drops to almost zero after 1 μs, the number of non-native contacts increases, resulting in the total stabilization of the complex. For the simulations with IGB = 1, 5, and 7, the number of H-bonds also increased toward the end of the simulation, while with IGB = 2, the number of H-bonds remained at the same level. On the other hand, the lowest number of total contacts as well as the least number of H-bonds are observed for the simulation using IGB = 8, which explains the lowest Δ*G* observed for this water model.

In summary, for the basic fibroblast factor–HP complex, any major change observed in the RMSD of the ligand corresponded to the Δ*G* variation for most of the water models. However, in the case of the simulation using the TIP3P water model, irrespective of the RMSD fluctuations, the Δ*G* of the system remains similar. For the simulations using TIP4P, TIP4PEw, and OPC water models, more deviation from the initial structure is found to be destabilizing in terms of Δ*G*. In the MD simulations using TIP5P and SPC/E water models, the more the deviations from the initial structure, the more the stabilization, and this agrees with the increased number of H-bonds and contacts. The simulations using implicit water models did not reveal any such relationship in the stability of the complex in terms of Δ*G* and the increase of RMSD; instead, most of them showed more stabilization of the complex irrespective of the RMSD, especially for the simulation with the IGB = 7 water model. Only the MD simulation with the IGB = 8 model showed less favorable Δ*G* corresponding to the significant structural changes observed.

### Cathepsin K–CS Complex

**Figure 8:**
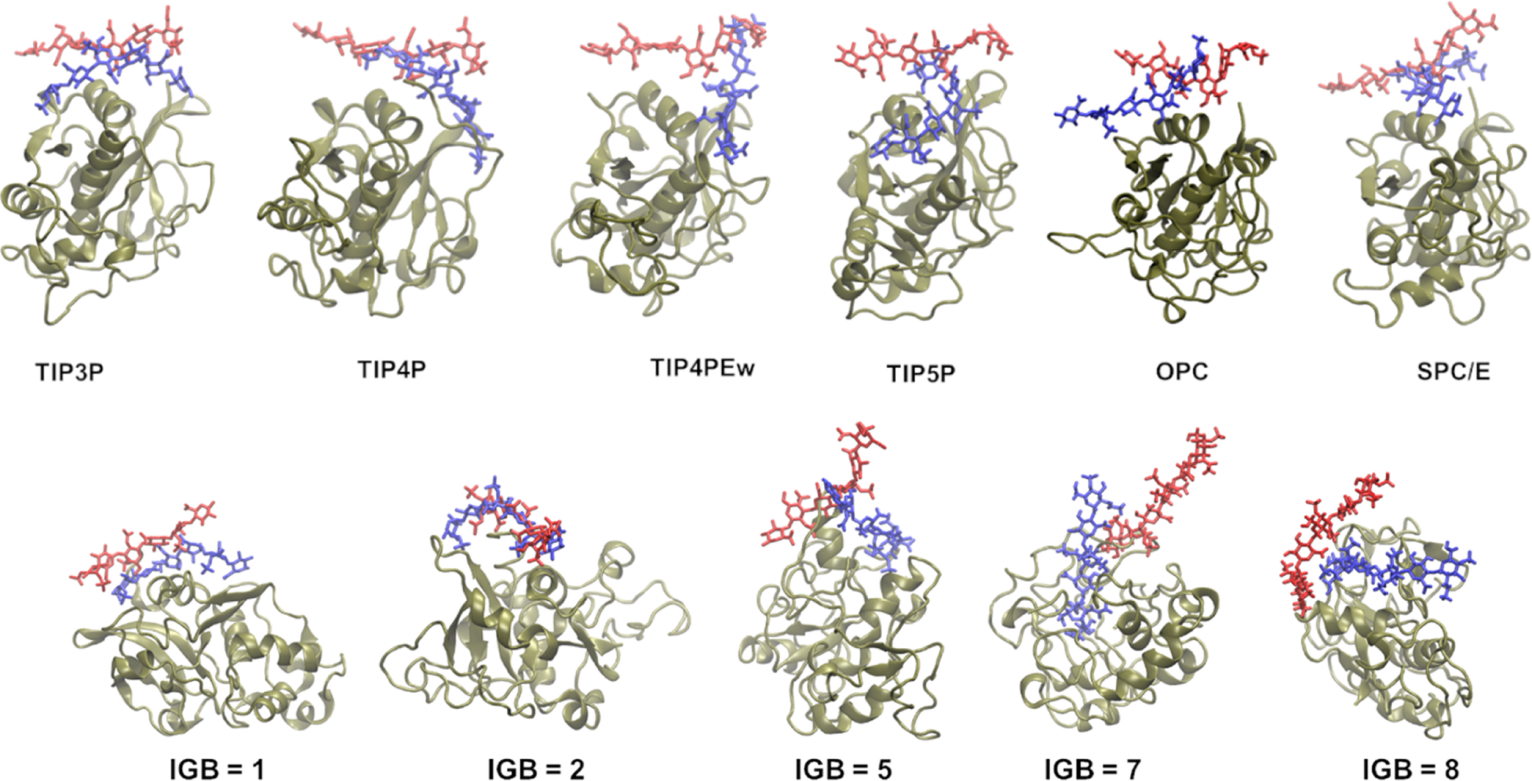
CS starting (red, licorice) and final (blue, licorice) poses in the complex with cathepsin K (tan, new cartoon). The initial structure corresponds to the 4N8W binding pose.

For the cathepsin K–CS complex, with the initial structure corresponding to the 4N8W binding pose, the complex is stable during the MD simulations using the water models TIP3P, OPC, IGB = 1, and IGB = 2, whereas with TIP4P, TIP4PEw, TIP5P, SPC/E, and IGB = 5, 7, and 8, the binding pose is essentially altered from the initial structure (Figure 8).

### Analysis of the Complex Stability

The predominant structural difference emerges in the directionality of the ligand (CS) in the specific binding pose. This can be considered as an outcome of the coexistence of two binding poses observed for the cathepsin K–CS complex experimentally (PDB IDs: 4N8W and 3C9E). Considering this, we calculated the RMSatD for the final structures of the simulation with references to both 4N8W and 3C9E structures. The RMSatD values provided in Table 1 clearly indicate that the final structure is more similar to the binding pose 3C9E in the MD simulations with most of the used water models except TIP4PEw. The highest RMSatDs are observed (for both 4N8W and 3C9E) in the simulations with the TIP4PEw model. Lower RMSatD values with reference to the 4N8W structure are obtained for the simulations with water models SPC/E, TIP3P, and TIP5P.

**Table 1:** RMSatD (Å) of the Cathepsin K–CS Complex with 4N8W and 3C9E

explicit water models			implicit water models		
model	4N8W	3C9E	model	4N8W	3C9E
TIP3P	8.5	6.4	IGB = 1	8.4	5.0
TIP4P	9.6	5.7	IGB = 2	9.1	3.4
TIP4PEw	10.6	10.8	IGB = 5	7.3	7.0
TIP5P	8.8	7.3	IGB = 7	16.4	14.1
OPC	10.3	5.0	IGB = 8	8.8	6.9
SPC/E	7.5	6.6			

As it was observed with the explicit solvents, the simulations of cathepsin K–CS with implicit solvent also result in different binding poses more similar to the 3C9E one. Compared to the simulations with explicit water models (Figures S5 and S6), those with implicit water result in significant changes in the protein structure (Figures S7 and S8). Among the simulations with implicit water models, more changes in the protein structure are observed for the ones with IGB = 7 and 8 (Table S2), which appear shortly after the start of the simulation (Figures S7 and S8). High RMSatD obtained for the MD simulation using IGB = 7 indicates significant differences from both crystal poses for this solvent model.

### Binding Free Energy Analysis

**Figure 9:**
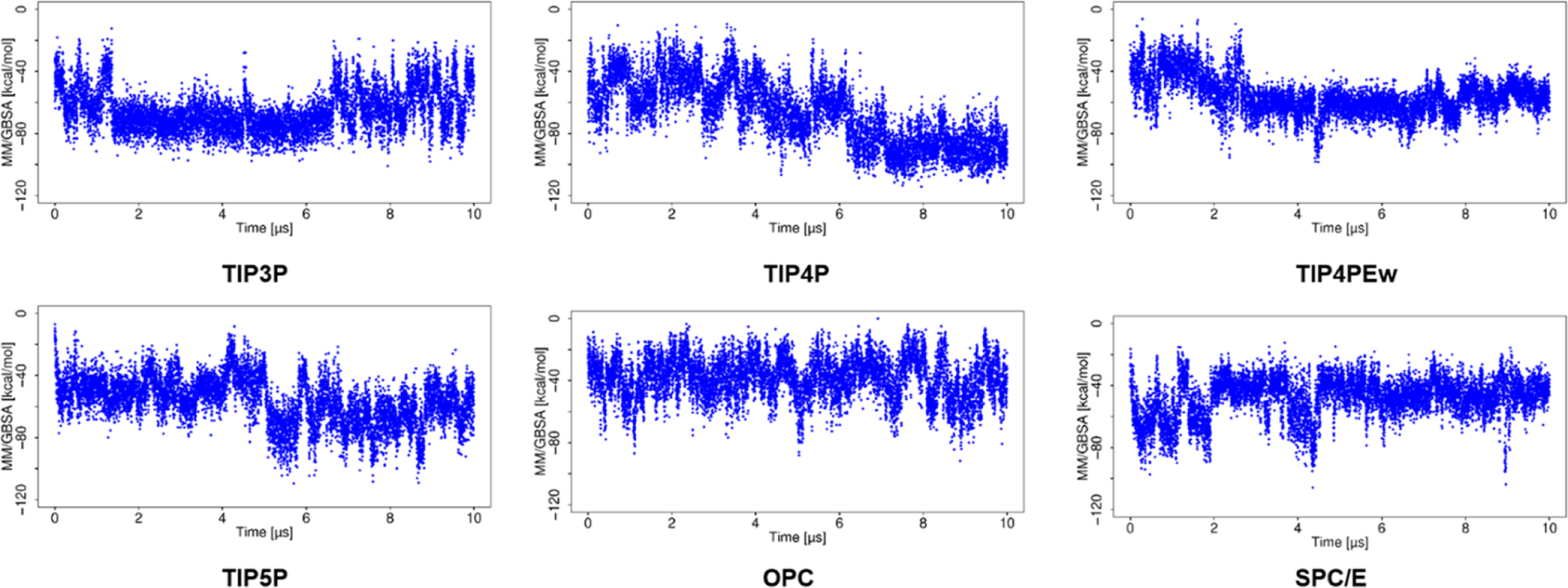
Δ*G* for the cathepsin K–CS complex in MD simulations with explicit water models.

**Figure 10:**
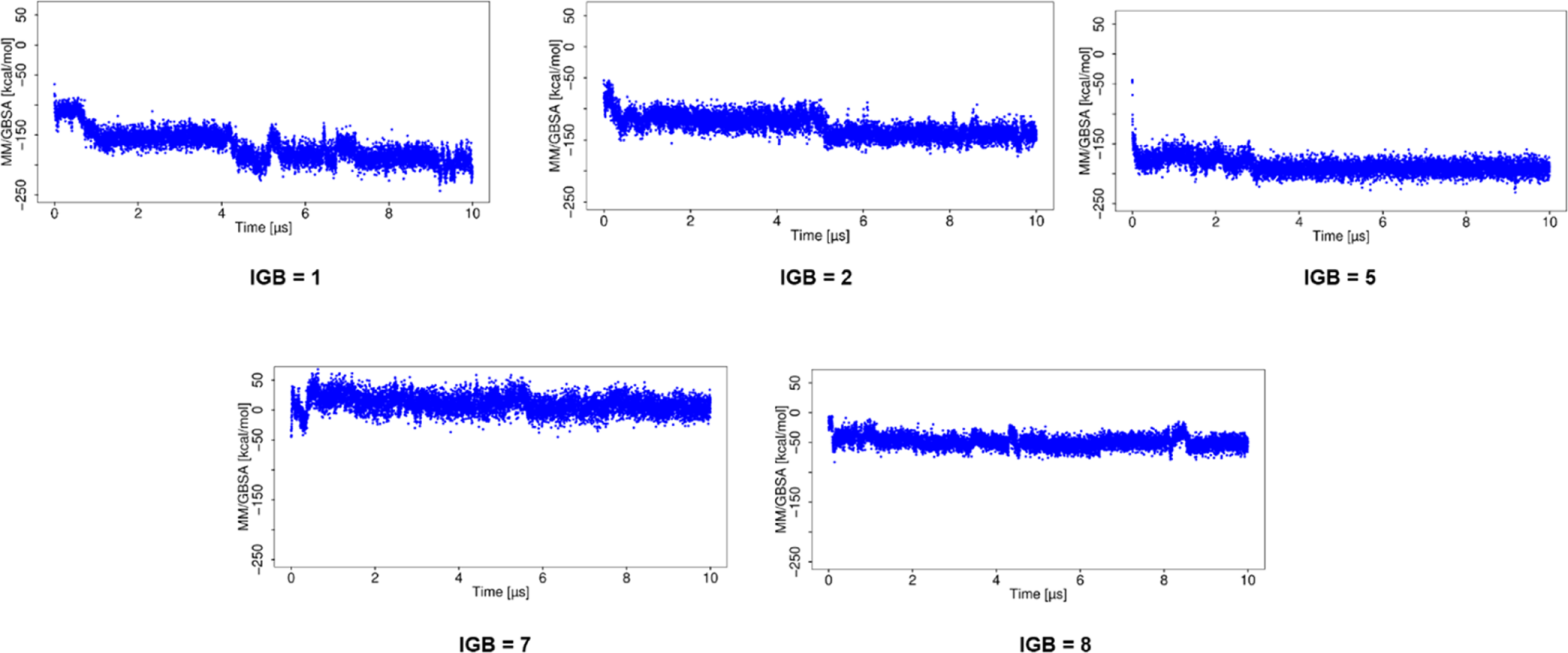
Δ*G* obtained for the cathepsin K–CS complex in MD simulations with implicit water models.

MM/GBSA analysis shows a more favorable Δ*G* corresponding to higher RMSD values (Figures 9 and 10). For the MD simulations using TIP4P, TIP4PEw, and TIP5P water models, as simulation extends, the RMSD (with respect to the starting structure) increases, and this structural deviation is found to have stabilizing effects on the complex reflected in more favorable Δ*G*. In the case of simulations using TIP3P, OPC, and SPC/E water models, the Δ*G* remains in a similar range with an average of −107.1 ± 12.9, −92.2 ± 15.3, and −115.9 ± 14.4 kcal/mol, respectively, independently of the structural changes. Among the simulations using implicit water models, slight RMSD increases correspond to minor unfavorable changes of Δ*G* for IGB = 1, 2, and 5. In the case of simulations with IGB = 7 and 8, more significant unfavorable changes are observed for Δ*G*. The variation in the Δ*G* for the simulation using the IGB = 7 water model indicates the relative instability of the complex compared to that observed in the simulations with other water models.

### Analysis of Contacts and H-Bonds

The analysis of the number of contacts shows that shortly after the beginning of the simulation, the number of native contacts declines to zero, and the number of non-native contacts increases, particularly in the case of simulations using TIP4P and TIP5P water models (Figure S9). A very similar trend can be observed for the number of H-bonds: particularly, the simulation with the TIP4P water model is marked with the significantly increased number of H-bonds with the course of the simulation (Figure S11). In the case of the MD simulations using implicit solvents, the number of contacts and H-bonds (Figures S10 and S12) also follows the same trend observed for RMSD and Δ*G*.

In the case of the moderately interacting cathepsin K–CS complex, the binding pose at the end of the simulation differs significantly from the starting one. The RMSatD analysis of the simulations of the cathepsin K–CS complex for most water models suggests a higher propensity toward the 3C9E pose than to the initial 4N8W pose. Similarly, the binding energy analysis showed that the final binding mode of the complex is more energetically favorable than that of the starting structure. The contact analysis showed that toward the end of the simulation, the number of native contacts almost declined to zero, with a substantial increase in the number of non-native contacts with respect to the 4N8W structure. The MD simulation using the TIP4P water model showed the most notable increase in the number of non-native contacts and the number of H-bonds. In the case of simulations with explicit water models, the Δ*G* changes correspond to the contact number changes, but for the implicit water model such a trend was not observed. Despite having a much larger number of non-native contacts than that observed in the simulations with other water models, the simulation using IGB = 7 does not show a significantly more favorable Δ*G*.

### CD44–HA Complex

**Figure 11:**
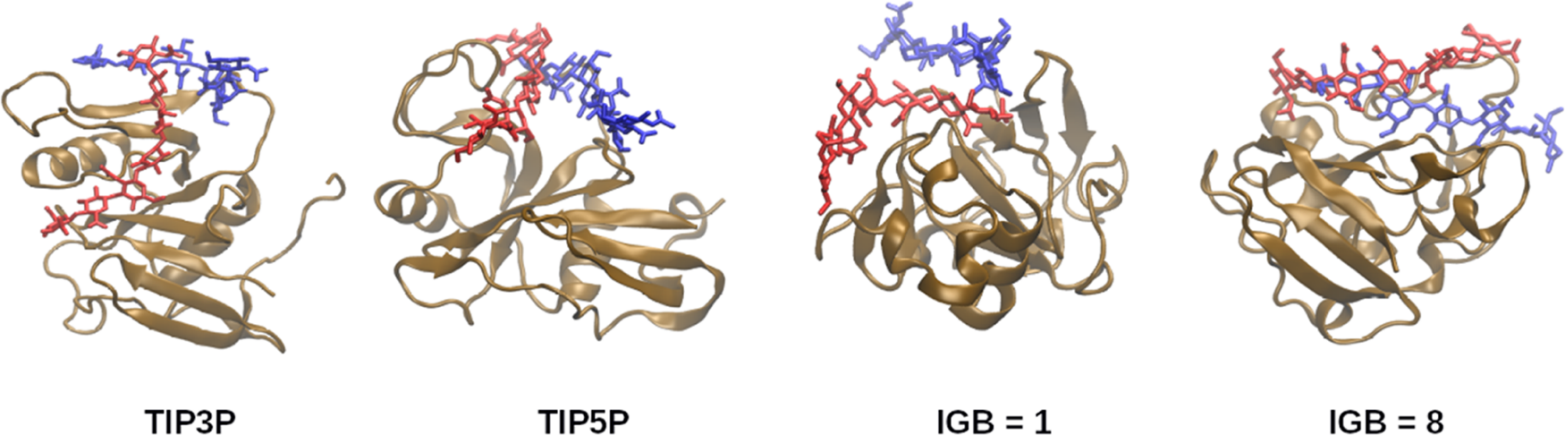
HA starting (red, licorice) and final (blue, licorice) poses in the binding site of CD44 (in brown, new cartoon).

In the case of the CD44–HA complex, MD simulations with most of the water models show that the complex dissociates after a few microseconds. A 10 μs long MD simulation of the CD44–HA complex did not lead to the dissociation only for TIP3P, TIP5P, and IGB = 1 and 8 water models. This may be because of the weaker affinity in this complex or the imperfections of the force fields used or due to the limitations of the X-ray structure. The CD44–HA complex is supposed to represent a case of multipose binding, as it was previously predicted by Vuorio et al.^62^Figure 11 shows the binding of the ligand (HA 8-mer) with CD44 in the simulations using TIP3P, TIP5P, and IGB = 1 and 8 water models.

### Analysis of the Complex Stability

**Figure 12:**
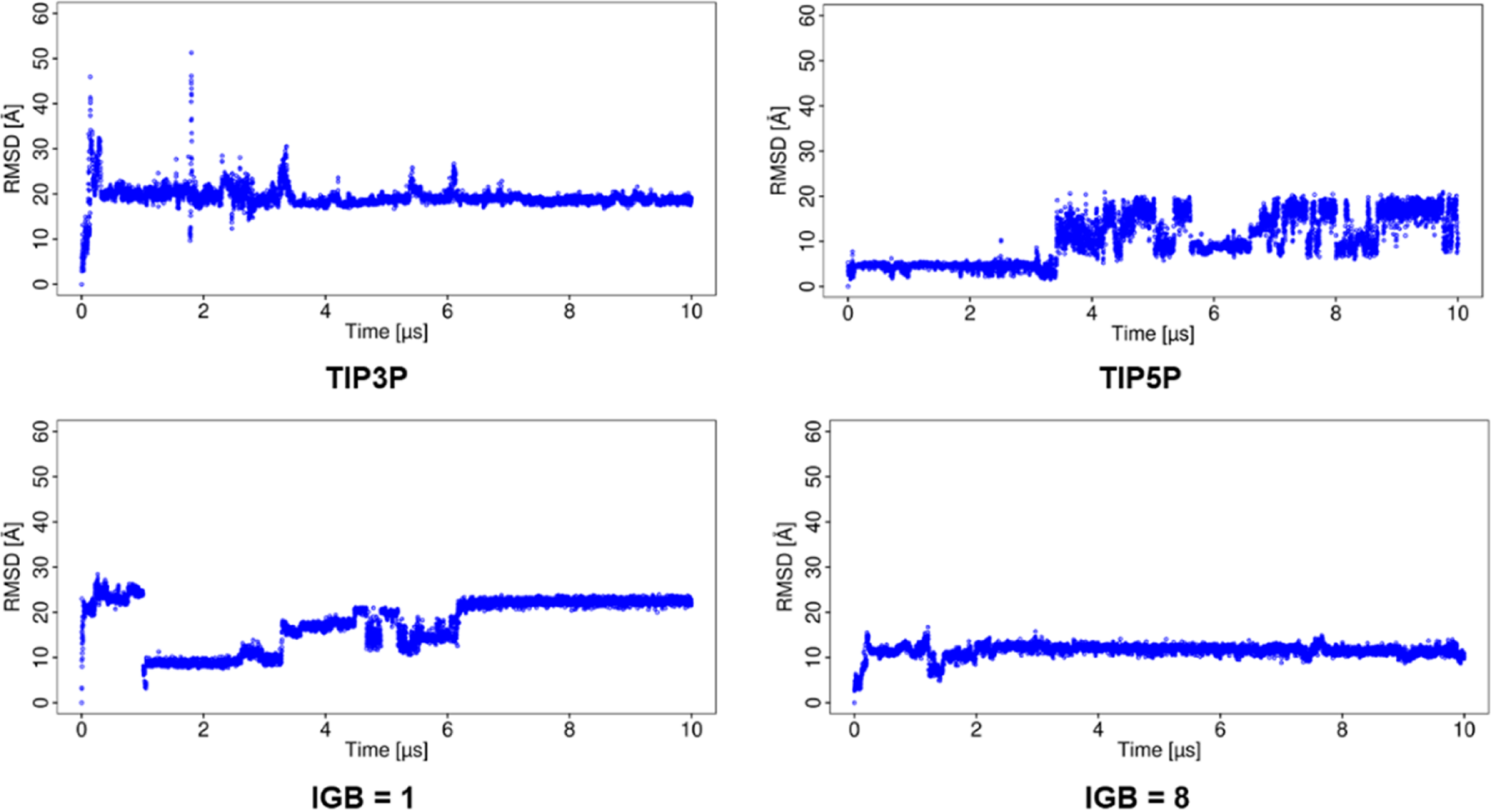
RMSD of the ligand in the CD44–HA complex.

RMSD with respect to the starting structure was calculated for the ligand (Figure 12) and the protein (Figure S13). The simulation using the TIP3P water model showed that the RMSD of the ligand increases to a remarkably high value (>20 Å) at the beginning of the simulation and remains in the same range. In the simulation using the TIP5P water model, the RMSD of the ligand was remarkably low (<5 Å) up to 3 μs and then increased to 10 Å remaining near this value. In the simulations with the implicit model IGB = 1, the RMSD of the ligand was even higher from the beginning of the simulation (>20 Å) while IGB = 8 showed a lower magnitude of RMSD for the ligand with an average of 11.5 ± 1.4 Å.

Compared to the ligand, the protein is found to be more stable in the simulations of both TIP3P and TIP5P water models with RMSD of 4.5 ± 0.6 and 3.8 ± 0.8 Å, respectively. In the MD simulations using implicit models IGB = 1 and IGB = 8, the protein showed slightly higher deviations from the starting structure than in the case of explicit models with RMSD of 6.0 ± 0.6 and 9.3 ± 1.6 Å, respectively. Thus, in comparison to other water models, the simulation with TIP5P showed the lowest deviations from the starting structure for the experimental binding pose (Table S3).

### Binding Free Energy Analysis

**Figure 13:**
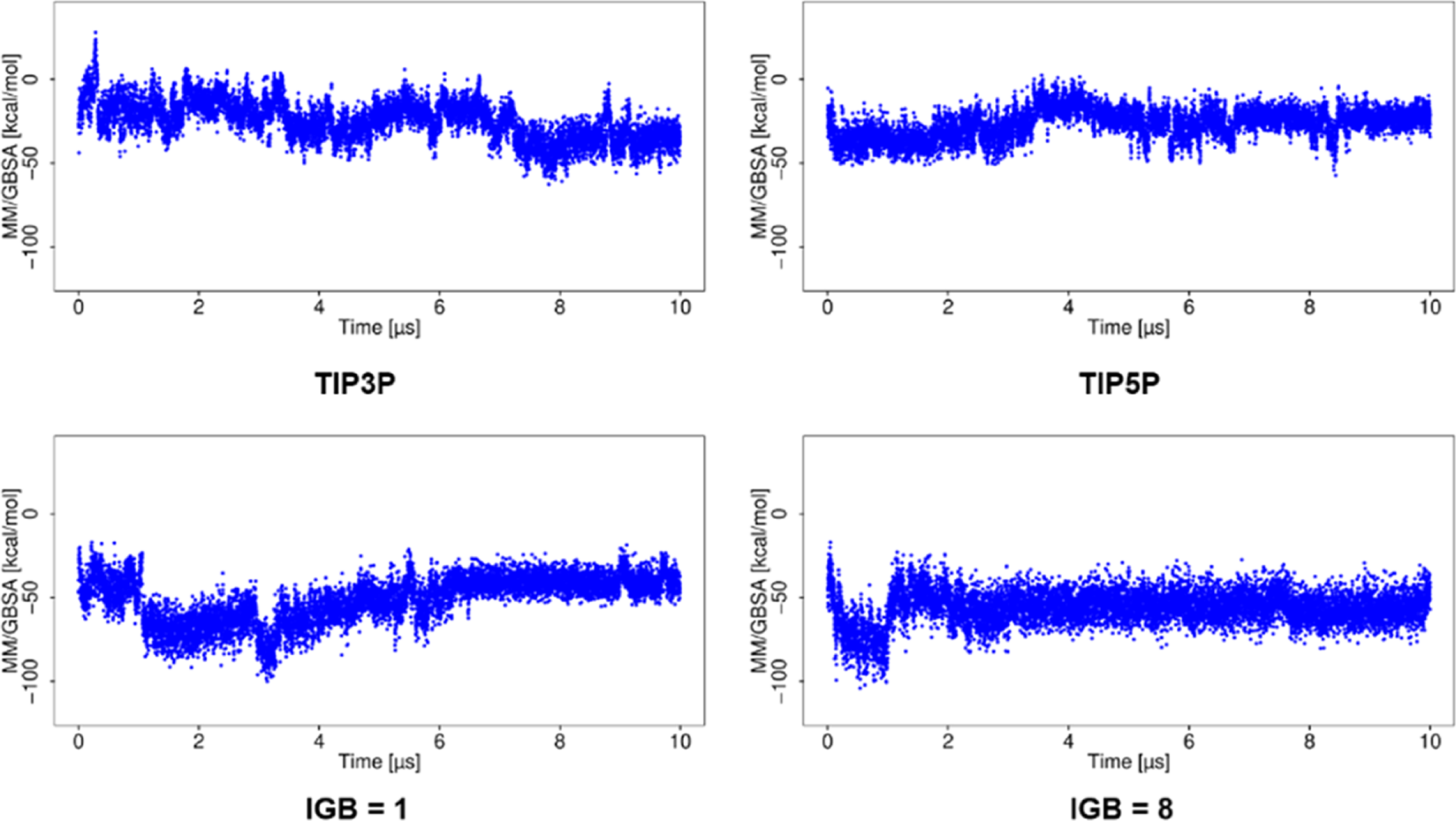
Δ*G* obtained for the CD44–HA complex.

The MM/GBSA analysis of the CD44–HA complex (Figure 13) reveals less favorable Δ*G* values for the interaction in this complex CD44 and HA compared to the other two complexes investigated in this study. This explains the observed dissociation of the CD44–HA complex in the simulations. Notably, during MD simulations employing the explicit water model TIP3P, increasing structural deviations (at the very beginning of the simulation) corresponded to a less favorable Δ*G*, and after this, when the RMSD converged steadily, more favorable Δ*G* was observed toward the end of the simulation. For the simulation using the TIP5P model, a significant increase in RMSD around the 4 μs correlates with less favorable Δ*G*. The simulations with implicit models also demonstrate less favorable Δ*G* values as RMSD increases.

### Analysis of Contacts and H-Bonds

**Figure 14:**
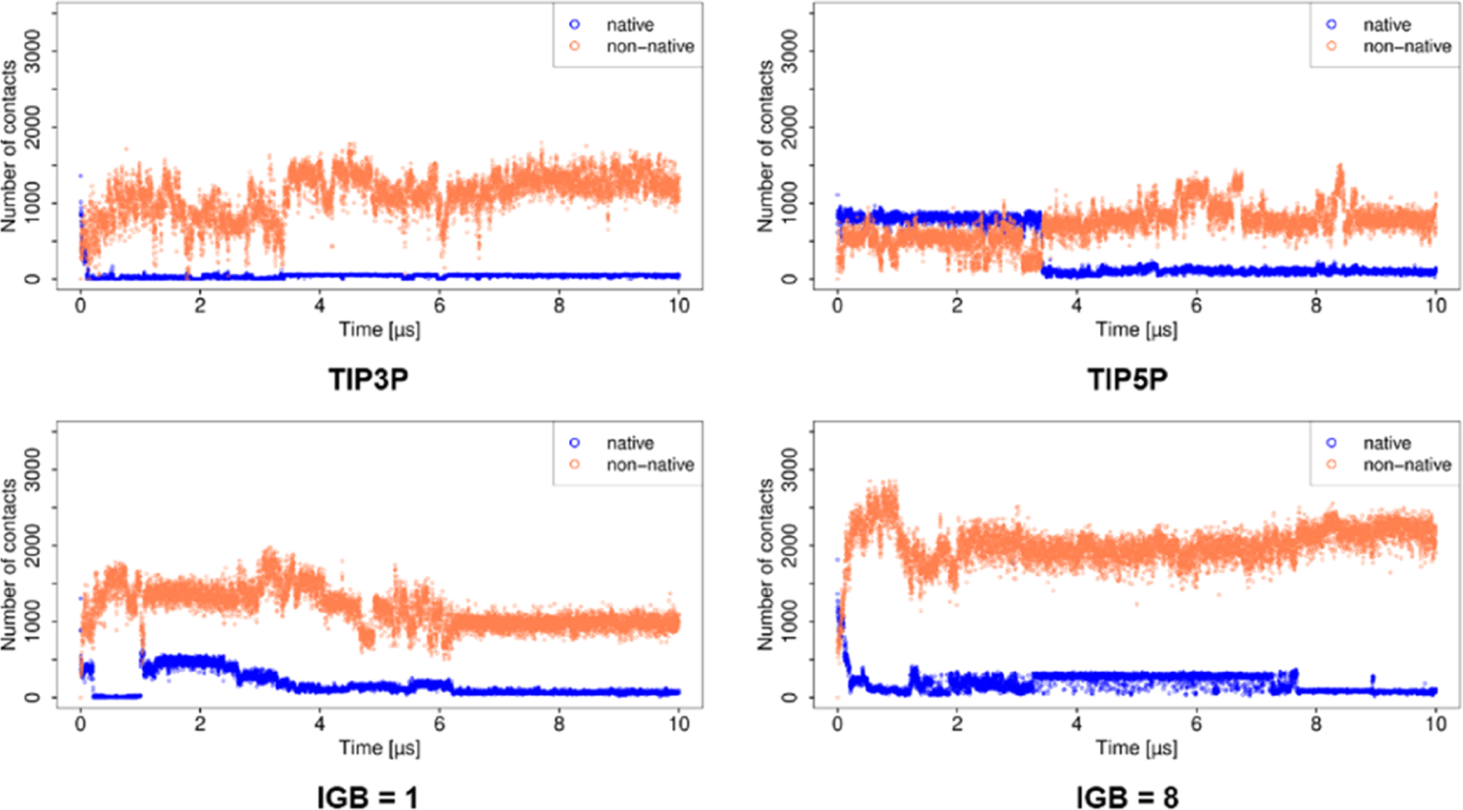
Number of contacts obtained for the CD44–HA complex.

For the simulation using the TIP3P water model, the number of native contacts declines to zero at the beginning of the simulation (Figure 14), corresponding to the increase in RMSD. Simultaneously, the number of non-native contacts increases, stabilizing the complex structure. In the simulation using the TIP5P water model, the number of native contacts drops to zero near the 4 μs, while the number of non-native contacts increases, reflecting converged binding energy. In the simulations with implicit water models, although the number of non-native contacts is remarkably high, the number of native contacts decreases to almost zero, whereas a high RMSD is observed for both the ligand and protein. The H-bonds (Figure S14) also showed the same trend as MM/GBSA and native contacts. Compared to the simulations using TIP3P, TIP5P, and IGB = 1, the simulation with the IGB = 8 model showed poor correspondence between the number of contacts or H-bonds to Δ*G*.

In the case of the weakly bound CD44–HA complex, the use of several water models led to the dissociation of HA from the CD44. No such dissociation was observed only for TIP3P, TIP5P, and IGB = 1 and 8. MD simulations with explicit water models yielded higher RMSD values and had less favorable Δ*G* values than the ones with the implicit water models. Similarly, a significantly higher number of non-native contacts is established with the implicit water models than with the explicit water models. Among the explicit water models, simulation using TIP3P is more stabilizing in terms of Δ*G* and in agreement with more H-bonds observed than that observed with the TIP5P water model (Figure S14). Although TIP5P showed stabilization effects comparable to that of TIP3P and IGB = 1 and 8 water models, the analysis of H-bonds showed that the MD simulation with TIP3P and IGB = 1 and 8 yielded a greater number of H-bonds than the one with TIP5P.

## Conclusions

The comparison of various water models in the MD simulations of three analyzed protein–GAG complexes shows that the type and model of the solvent have a substantial effect on their MD simulation results. The stronger the binding interaction of the protein–GAG complex, the lesser the effect of the water models on the stability and energetics of the complex. In general, the study showed that the structural features and energetics of the complexes can be better understood in the simulations using explicit water models. The trends observed with the complex stability in terms of RMSD and MM/GBSA binding free energies were consistent with the observations from the analysis of the contacts and H-bonds in all three complexes.

Our investigation of three representative protein–ligand systems has provided valuable insights into the interplay between their dynamics and energetics, revealing the diverse effects of water models on protein–GAG interactions. The strongly bound basic fibroblast factor–HP complex exhibited stability, with the TIP3P water model displaying unique behavior. The use of the TIP4P, TIP4PEw, and OPC models destabilized the interactions with increased structural deviations, while TIP5P and SPC/E showed enhanced stabilization upon structural changes. Implicit water models generally favored stabilization during the MD simulation, especially IGB = 7, but IGB = 8 yielded significantly less favorable Δ*G*. The change in RMSD or Δ*G* or number of contacts/H-bonds of the moderately interacting cathepsin K–CS complex were specific for MD simulations using different explicit water models, while simulations using implicit water models showed similar trends, with a notable change in binding mode observed at the end of the simulation. Most importantly, the use of all of the water models for this complex showed that the binding pose at the end of the simulation is more similar to that in 3C9E than to the 4N8W crystal structure used as the starting conformation. In the case of the relatively unstable CD44–HA complex, no dissociation was observed during simualtions with a few water models, highlighting the critical role of water models in simulating complex dynamics.

This systematic investigation, comparing the impact of different water models on the binding characteristics and stability of three representative and well-characterized protein–GAG systems, clearly highlights the notable advantages of explicit water models over their implicit counterparts. The MD simulations using explicit water models exhibited a remarkable ability to capture distinct features in terms of RMSD, Δ*G*, and the number of contacts and H-bonds. In contrast, MD simulations with different implicit water models yielded similar outcomes across various metrics used in this study. In particular, the simulations with implicit water models displayed reduced variations and fluctuations of the analyzed descriptors for the CD44–HA complex with a low binding affinity. At the same time, our analysis indicated a tendency of implicit water models to overestimate the binding energy and the number of non-native contacts. This convincing evidence highlights the superior performance of explicit water models in representing the intricate dynamics and stability profiles of protein–GAG interactions. The observed variations in various descriptors across different simulation conditions emphasize the importance of the chosen water model for the MD simulation of protein–GAG interactions, thereby highlighting the discrete nature of their underlying dynamics for accurate analysis and design of the GAG-based drugs.

## Data Availability

All of the data underlying this study are available in the manuscript and its Supporting Information files. Apart from that, all of the data (MD simulations) were obtained using the AMBER suite (Amber20 and AmberTools). The data were then analyzed using R (statistics and plots), VMD (visualization of the obtained trajectories), and GIMP (figure preparation). All software except for the AMBER suite is free of charge. AMBER software can be obtained from http://ambermd.org/GetAmber.php. R can be downloaded from https://www.r-project.org/. GIMP can be downloaded from https://www.gimp.org/. VMD can be downloaded from http://www.ks.uiuc.edu/Research/vmd/.

## Supporting Information Available

Graphical representation of different parameters analyzed in this work: RMSD, number of H-bonds, and number of native and non-native contacts obtained for the protein–GAG complexes in MD simulations using different water models used in this study; the tables of RMSD (mean and standard deviation) of the ligand and protein for the protein–GAG complexes in MD simulations using various water models (PDF)
Initial coordinates; topology files; and input files used to generate MD trajectories (ZIP)

## References

[ref1] Varki A.; Cummings R. D.; Esko J. D.; Stanley P.; Hart G. W.; Aebi M.; Mohnen D.; Kinoshita T.; Packer N. H.; Prestegard J. H. Study Guide. In Essentials of Glycobiology [Internet], 4th ed.; Cold Spring Harbor Laboratory Press: New York, 2022. 35536922

[ref2] Chen J.; Sun T.; You Y.; Wu B.; Wang X.; Wu J. Proteoglycans and glycosaminoglycans in stem cell homeostasis and bone tissue regeneration. Front. Cell Dev. Biol. 2021, 9, 760532 10.3389/fcell.2021.760532. 34917612 PMC8669051

[ref3] Wigén J.; Elowsson-Rendin L.; Karlsson L.; Tykesson E.; Westergren-Thorsson G. Glycosaminoglycans: a link between development and regeneration in the lung. Stem Cells Dev. 2019, 28, 823–832. 10.1089/scd.2019.0009. 31062651

[ref4] Dieckmann C.; Renner R.; Milkova L.; Simon J. C. Regenerative medicine in dermatology: biomaterials, tissue engineering, stem cells, gene transfer and beyond. Exp. Dermatol. 2010, 19, 697–706. 10.1111/j.1600-0625.2010.01087.x. 20545761

[ref5] Paganini C.; Costantini R.; Superti-Furga A.; Rossi A. Bone and connective tissue disorders caused by defects in glycosaminoglycan biosynthesis: a panoramic view. FEBS J. 2019, 286, 3008–3032. 10.1111/febs.14984. 31286677

[ref6] Salbach J.; Rachner T. D.; Rauner M.; Hempel U.; Anderegg U.; Franz S.; Simon J.-C.; Hofbauer L. C. Regenerative potential of glycosaminoglycans for skin and bone. J. Mol. Med. 2012, 90, 625–635. 10.1007/s00109-011-0843-2. 22187113

[ref7] Prydz K. Determinants of glycosaminoglycan (GAG) structure. Biomolecules 2015, 5, 2003–2022. 10.3390/biom5032003. 26308067 PMC4598785

[ref8] Sasisekharan R.; Venkataraman G. Heparin and heparan sulfate: biosynthesis, structure and function. Curr. Opin. Chem. Biol. 2000, 4, 626–631. 10.1016/S1367-5931(00)00145-9. 11102866

[ref9] Mizumoto S.; Yamada S.; Sugahara K. Molecular interactions between chondroitin–dermatan sulfate and growth factors/receptors/matrix proteins. Curr. Opin. Struct. Biol. 2015, 34, 35–42. 10.1016/j.sbi.2015.06.004. 26164146

[ref10] Funderburgh J. L. MINI REVIEW Keratan sulfate: structure, biosynthesis, and function. Glycobiology 2000, 10, 951–958. 10.1093/glycob/10.10.951. 11030741

[ref11] Almond A. Hyaluronan. Cell. Mol. Life Sci. 2007, 64, 1591–1596. 10.1007/s00018-007-7032-z. 17502996 PMC11136421

[ref12] Soares P. A.; Queiroz I. N.; Pomin V. H. NMR structural biology of sulfated glycans. J. Biomol. Struct. Dyn. 2017, 35, 1069–1084. 10.1080/07391102.2016.1171165. 27166778

[ref13] Habuchi H.; Habuchi O.; Kimata K. Sulfation pattern in glycosaminoglycan: does it have a code?. Glycoconjugate J. 2004, 21, 47–52. 10.1023/B:GLYC.0000043747.87325.5e. 15467398

[ref14] Kogut M. M.; Marcisz M.; Samsonov S. A. Modeling glycosaminoglycan–protein complexes. Curr. Opin. Struct. Biol. 2022, 73, 102332 10.1016/j.sbi.2022.102332. 35152187

[ref15] Perez S.; Makshakova O.; Angulo J.; Bedini E.; Bisio A.; de Paz J. L.; Fadda E.; Guerrini M.; Hricovini M.; Hricovini M.; et al. Glycosaminoglycans: what remains to be deciphered?. JACS Au 2023, 3, 628–656. 10.1021/jacsau.2c00569. 37006755 PMC10052243

[ref16] Derler R.; Gesslbauer B.; Weber C.; Strutzmann E.; Miller I.; Kungl A. Glycosaminoglycan-mediated downstream signaling of CXCL8 binding to endothelial cells. Int. J. Mol. Sci. 2017, 18, 2605 10.3390/ijms18122605. 29207576 PMC5751208

[ref17] Hudalla G. A.; Murphy W. L. Biomaterials that regulate growth factor activity via bioinspired interactions. Adv. Funct. Mater. 2011, 21, 1754–1768. 10.1002/adfm.201002468. 21921999 PMC3171147

[ref18] Karamanos N. K.; Piperigkou Z.; Theocharis A. D.; Watanabe H.; Franchi M.; Baud S.; Brezillon S.; Götte M.; Passi A.; Vigetti D.; et al. Proteoglycan chemical diversity drives multifunctional cell regulation and therapeutics. Chem. Rev. 2018, 118, 9152–9232. 10.1021/acs.chemrev.8b00354. 30204432

[ref19] Moustakas A.; Souchelnytskyi S.; Heldin C.-H. Smad regulation in TGF-β signal transduction. J. Cell Sci. 2001, 114, 4359–4369. 10.1242/jcs.114.24.4359. 11792802

[ref20] Penk A.; Baumann L.; Huster D.; Samsonov S. A. NMR and molecular modeling reveal specificity of the interactions between CXCL14 and glycosaminoglycans. Glycobiology 2019, 29, 715–725. 10.1093/glycob/cwz047. 31264681

[ref21] Samantray S.; Olubiyi O. O.; Strodel B. The Influences of Sulphation, Salt Type, and Salt Concentration on the Structural Heterogeneity of Glycosaminoglycans. Int. J. Mol. Sci. 2021, 22, 11529 10.3390/ijms222111529. 34768961 PMC8583755

[ref22] Imberty A.; Lortat-Jacob H.; Pérez S. Structural view of glycosaminoglycan–protein interactions. Carbohydr. Res. 2007, 342, 430–439. 10.1016/j.carres.2006.12.019. 17229412

[ref23] Nagarajan B.; Holmes S. G.; Sankaranarayanan N. V.; Desai U. R. Molecular dynamics simulations to understand glycosaminoglycan interactions in the free-and protein-bound states. Curr. Opin. Struct. Biol. 2022, 74, 102356 10.1016/j.sbi.2022.102356. 35306321 PMC9189024

[ref24] Petitou M.; Casu B.; Lindahl U. 1976–1983, a critical period in the history of heparin: the discovery of the antithrombin binding site. Biochimie 2003, 85, 83–89. 10.1016/S0300-9084(03)00078-6. 12765778

[ref25] Sepuru K. M.; Nagarajan B.; Desai U. R.; Rajarathnam K. Structural basis, stoichiometry, and thermodynamics of binding of the chemokines KC and MIP2 to the glycosaminoglycan heparin. J. Biol. Chem. 2018, 293, 17817–17828. 10.1074/jbc.RA118.004866. 30257866 PMC6240865

[ref26] Foley B. L.; Tessier M. B.; Woods R. J. Carbohydrate force fields. Wiley Interdiscip. Rev.: Comput. Mol. Sci. 2012, 2, 652–697. 10.1002/wcms.89. 25530813 PMC4270206

[ref27] Kerzmann A.; Fuhrmann J.; Kohlbacher O.; Neumann D. BALLDock/SLICK: a new method for protein-carbohydrate docking. J. Chem. Inf. Model. 2008, 48, 1616–1625. 10.1021/ci800103u. 18646839

[ref28] Kerzmann A.; Neumann D.; Kohlbacher O. SLICK—Scoring and Energy Functions for Protein–Carbohydrate Interactions. J. Chem. Inf. Model. 2006, 46, 1635–1642. 10.1021/ci050422y. 16859295

[ref29] Taroni C.; Jones S.; Thornton J. M. Analysis and prediction of carbohydrate binding sites. Protein Eng., Des. Sel. 2000, 13, 89–98. 10.1093/protein/13.2.89. 10708647

[ref30] Samsonov S. A.; Pisabarro M. T. Computational analysis of interactions in structurally available protein–glycosaminoglycan complexes. Glycobiology 2016, 26, 850–861. 10.1093/glycob/cww055. 27496767

[ref31] Samsonov S.; Teyra J.; Pisabarro M. T. A molecular dynamics approach to study the importance of solvent in protein interactions. Proteins 2008, 73, 515–525. 10.1002/prot.22076. 18452208

[ref32] Samsonov S. A.; Gehrcke J.-P.; Pisabarro M. T. Flexibility and explicit solvent in molecular-dynamics-based docking of protein–glycosaminoglycan systems. J. Chem. Inf. Model 2014, 54, 582–592. 10.1021/ci4006047. 24479827

[ref33] Möbius K.; Nordsieck K.; Pichert A.; Samsonov S. A.; Thomas L.; Schiller J.; Kalkhof S.; Teresa Pisabarro M.; Beck-Sickinger A. G.; Huster D. Investigation of lysine side chain interactions of interleukin-8 with heparin and other glycosaminoglycans studied by a methylation-NMR approach. Glycobiology 2013, 23, 1260–1269. 10.1093/glycob/cwt062. 23982278

[ref34] Nguyen H.; Roe D. R.; Simmerling C. Improved generalized born solvent model parameters for protein simulations. J. Chem. Theory Comput. 2013, 9, 2020–2034. 10.1021/ct3010485. 25788871 PMC4361090

[ref35] Onufriev A. V.; Case D. A. Generalized Born implicit solvent models for biomolecules. Annu. Rev. Biophys. 2019, 48, 275–296. 10.1146/annurev-biophys-052118-115325. 30857399 PMC6645684

[ref36] Lang E. J. M.; Baker E. G.; Woolfson D. N.; Mulholland A. J. Generalized Born implicit solvent models do not reproduce secondary structures of de novo designed Glu/Lys peptides. J. Chem. Theory Comput. 2022, 18, 4070–4076. 10.1021/acs.jctc.1c01172. 35687842 PMC9281390

[ref37] Marcisz M.; Gaardløs M.; Bojarski K. K.; Siebenmorgen T.; Zacharias M.; Samsonov S. A. Explicit solvent repulsive scaling replica exchange molecular dynamics (RS-REMD) in molecular modeling of protein-glycosaminoglycan complexes. J. Comput. Chem. 2022, 43, 1633–1640. 10.1002/jcc.26965. 35796487

[ref38] Nagarajan B.; Sankaranarayanan N. V.; Patel B. B.; Desai U. R. A molecular dynamics-based algorithm for evaluating the glycosaminoglycan mimicking potential of synthetic, homogenous, sulfated small molecules. PLoS One 2017, 12, e0171619 10.1371/journal.pone.0171619. 28182755 PMC5300208

[ref39] Sapay N.; Cabannes E.; Petitou M.; Imberty A. Molecular modeling of the interaction between heparan sulfate and cellular growth factors: bringing pieces together. Glycobiology 2011, 21, 1181–1193. 10.1093/glycob/cwr052. 21572110

[ref40] Izadi S.; Anandakrishnan R.; Onufriev A. V. Building water models: a different approach. J. Phys. Chem. Lett. 2014, 5, 3863–3871. 10.1021/jz501780a. 25400877 PMC4226301

[ref41] Izadi S.; Onufriev A. V. Accuracy limit of rigid 3-point water models. J. Chem. Phys. 2016, 145, 074501 10.1063/1.4960175. 27544113 PMC4991989

[ref42] Verli H.; Guimarães J. A. Molecular dynamics simulation of a decasaccharide fragment of heparin in aqueous solution. Carbohydr. Res. 2004, 339, 281–290. 10.1016/j.carres.2003.09.026. 14698886

[ref43] Neamtu A.; Tamba B.; Patras X. Molecular dynamics simulations of chondroitin sulfate in explicit solvent: Point charge water models compared. Cellul. Chem. Technol. 2013, 47, 191–202.

[ref44] Marcisz M.; Samsonov S. A. Solvent Model Benchmark for Molecular Dynamics of Glycosaminoglycans. J. Chem. Inf. Model. 2023, 63, 2147–2157. 10.1021/acs.jcim.2c01472. 36989082 PMC10091405

[ref45] Almond A.; Sheehan J. K. Glycosaminoglycan conformation: do aqueous molecular dynamics simulations agree with x-ray fiber diffraction?. Glycobiology 2000, 10, 329–338. 10.1093/glycob/10.3.329. 10704532

[ref46] Case D. A.; Belfon K.; Ben-Shalom I. Y.; Brozell S. R.; Cerutti D. S.; Cheatham T. E.; Cruzeiro V. W. D.; Darden T. A.; Duke R. E.; Giambasu G.; Gilson M. K.; Gohlke H.; Goetz A. W.; Harris R.; Izadi S.; Izmailov S. A.; Kasavajhala K.; Kovalenko A.; Krasny R.; Kurtzman T.; Lee T. S.; LeGrand S.; Li P.; Lin C.; Liu J.; Luchko T.; Luo R.; Man V.; Merz K. M.; Miao Y.; Mikhailovskii O.; Monard G.; Nguyen H.; Onufriev A.; Pan F.; Pantano F.; Qi R.; Roe D. R.; Roitberg A.; Sagui C.; Schott-Verdugo S.; Shen J.; Simmerling C. L.; Skrynnikov N. R.; Smith J.; Swails K.; Walker R. C.; Wang J.; Wilson L.; Wolf R. M.; Wu X.; Xiong Y.; Xue Y.; York D. M.; Kollman P. A. AMBER 2020; University of California: San Francisco, 2020.

[ref47] Onufriev A.; Case D. A.; Bashford D. Effective Born radii in the generalized Born approximation: the importance of being perfect. J. Comput. Chem. 2002, 23, 1297–1304. 10.1002/jcc.10126. 12214312

[ref48] Jorgensen W. L.; Chandrasekhar J.; Madura J. D.; Impey R. W.; Klein M. L. Comparison of simple potential functions for simulating liquid water. J. Chem. Phys. 1983, 79, 926–935. 10.1063/1.445869.

[ref49] Neria E.; Fischer S.; Karplus M. Simulation of activation free energies in molecular systems. J. Chem. Phys. 1996, 105, 1902–1921. 10.1063/1.472061.

[ref50] Berendsen H. J. C.; Grigera J. R.; Straatsma T. P. The missing term in effective pair potentials. J. Phys. Chem. A 1987, 91, 6269–6271. 10.1021/j100308a038.

[ref51] Horn H. W.; Swope W. C.; Pitera J. W.; Madura J. D.; Dick T. J.; Hura G. L.; Head-Gordon T. Development of an improved four-site water model for biomolecular simulations: TIP4P-Ew. J. Chem. Phys. 2004, 120, 9665–9678. 10.1063/1.1683075. 15267980

[ref52] Mahoney M. W.; Jorgensen W. L. A five-site model for liquid water and the reproduction of the density anomaly by rigid, nonpolarizable potential functions. J. Chem. Phys. 2000, 112, 8910–8922. 10.1063/1.481505.

[ref53] Hawkins G. D.; Cramer C. J.; Truhlar D. G. Pairwise solute descreening of solute charges from a dielectric medium. Chem. Phys. Lett. 1995, 246, 122–129. 10.1016/0009-2614(95)01082-K.

[ref54] Onufriev A.; Bashford D.; Case D. A. Modification of the generalized Born model suitable for macromolecules. J. Phys. Chem. B 2000, 104, 3712–3720. 10.1021/jp994072s.

[ref55] Onufriev A.; Bashford D.; Case D. A. Exploring protein native states and large-scale conformational changes with a modified generalized born model. Proteins 2004, 55, 383–394. 10.1002/prot.20033. 15048829

[ref56] Mongan J.; Simmerling C.; McCammon J. A.; Case D. A.; Onufriev A. Generalized Born model with a simple, robust molecular volume correction. J. Chem. Theory Comput. 2007, 3, 156–169. 10.1021/ct600085e. 21072141 PMC2975579

[ref57] Ryckaert J.-P.; Ciccotti G.; Berendsen H. J. Numerical integration of the cartesian equations of motion of a system with constraints: molecular dynamics of n-alkanes. J. Comput. Phys. 1977, 23, 327–341. 10.1016/0021-9991(77)90098-5.

[ref58] Darden T.; York D.; Pedersen L. Particle mesh Ewald: An N· log (N) method for Ewald sums in large systems. J. Chem. Phys. 1993, 98, 10089–10092. 10.1063/1.464397.

[ref59] Roe D. R.; Cheatham T. E. III PTRAJ and CPPTRAJ: software for processing and analysis of molecular dynamics trajectory data. J. Chem. Theory Comput. 2013, 9, 3084–3095. 10.1021/ct400341p. 26583988

[ref60] Core_Team_(2013). R: A Language and Environment for Statistical Computing; R. R Development Core Team, 2013.

[ref61] Humphrey W.; Dalke A.; Schulten K. VMD: visual molecular dynamics. J. Mol. Graphics 1996, 14, 33–38. 10.1016/0263-7855(96)00018-5. 8744570

[ref62] Vuorio J.; Vattulainen I.; Martinez-Seara H. Atomistic fingerprint of hyaluronan–CD44 binding. PLoS Comput. Biol. 2017, 13, e1005663 10.1371/journal.pcbi.1005663. 28715483 PMC5549728

