# Supplementary material for: Benchmarking Water Models in Molecular Dynamics of Protein–Glycosaminoglycan Complexes
Source: J Chem Inf Model. 2024 Feb 27;64(5):1691–703. doi: 10.1021/acs.jcim.4c00030 (PMC10934818; doi:10.1021/acs.jcim.4c00030)
Supplement: Supplementary file 1 — ci4c00030_si_001.pdf [file ci4c00030_si_001.pdf]

Supporting Information

Benchmarking Water Models in Molecular Dynamics of Protein-Glycosaminoglycan Complexes

Sebastian Anila[1] and Sergey A. Samsonov[1]*

[1]Faculty of Chemistry, University of Gdańsk, ul. Wita Stwosza 63, 80-308 Gdańsk, Poland.

**Corresponding authors:* Sergey A. Samsonov (sergey.samsonov@ug.edu.pl)

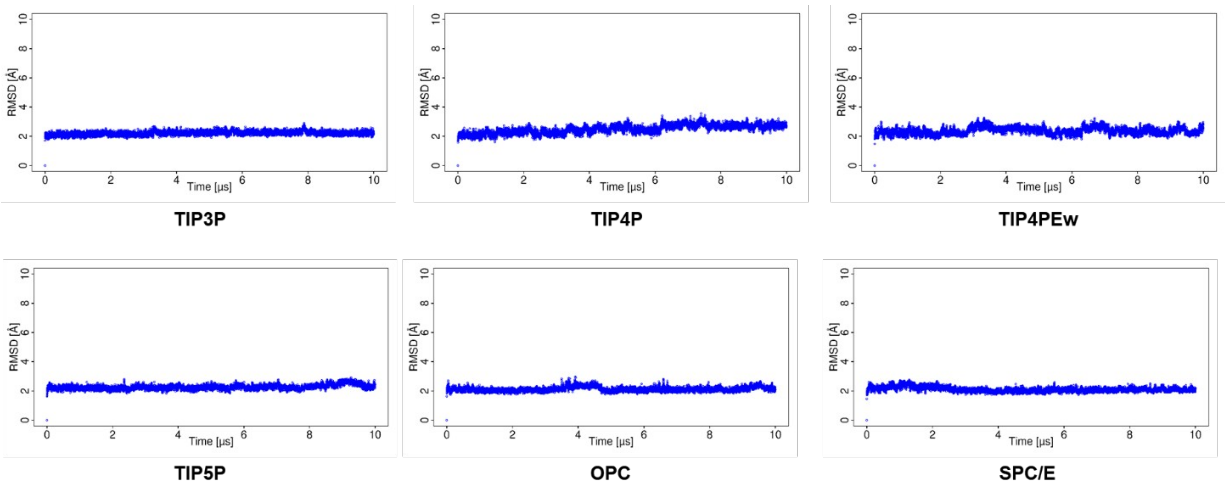

Figure S1. RMSD of the protein for Basic Fibroblast Factor-HP complex in MD simulations using explicit water models.

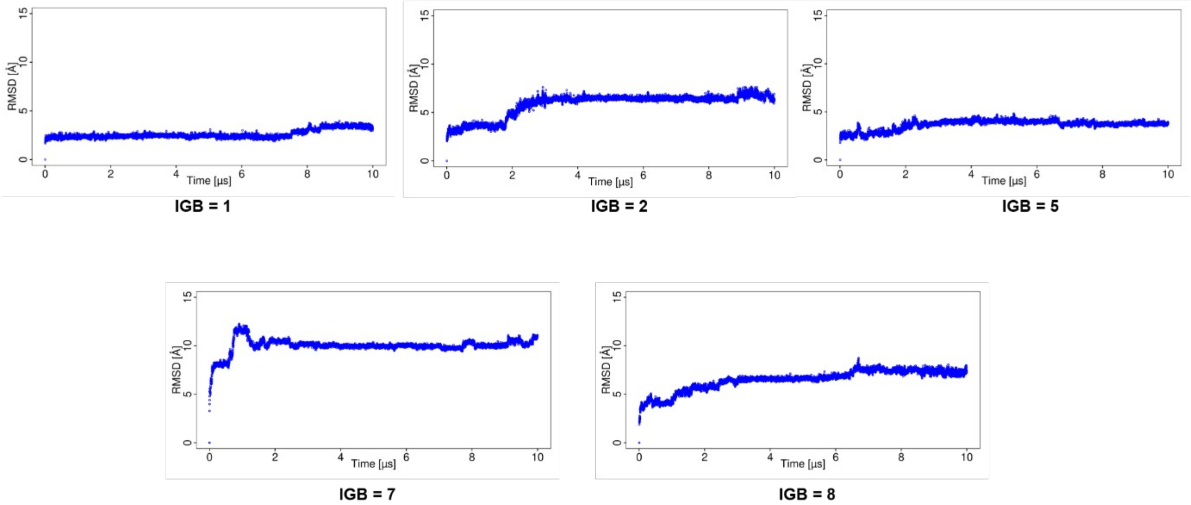

Figure S2. RMSD of the protein for Basic Fibroblast Factor-HP complex in MD simulations using implicit water models.

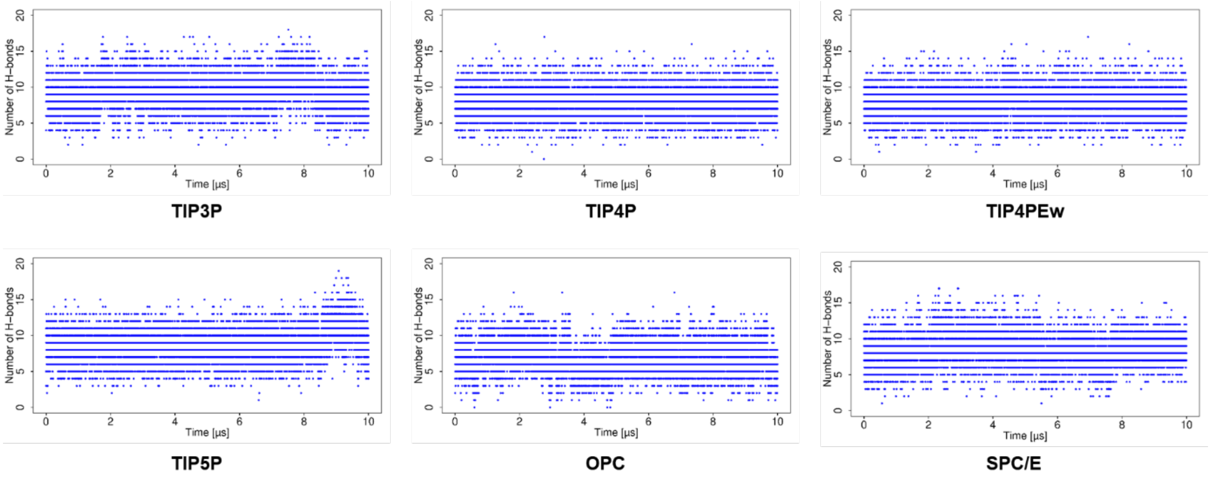

Figure S3. Number of H-bonds obtained for Basic Fibroblast Factor-HP complex in MD simulations using explicit water models.

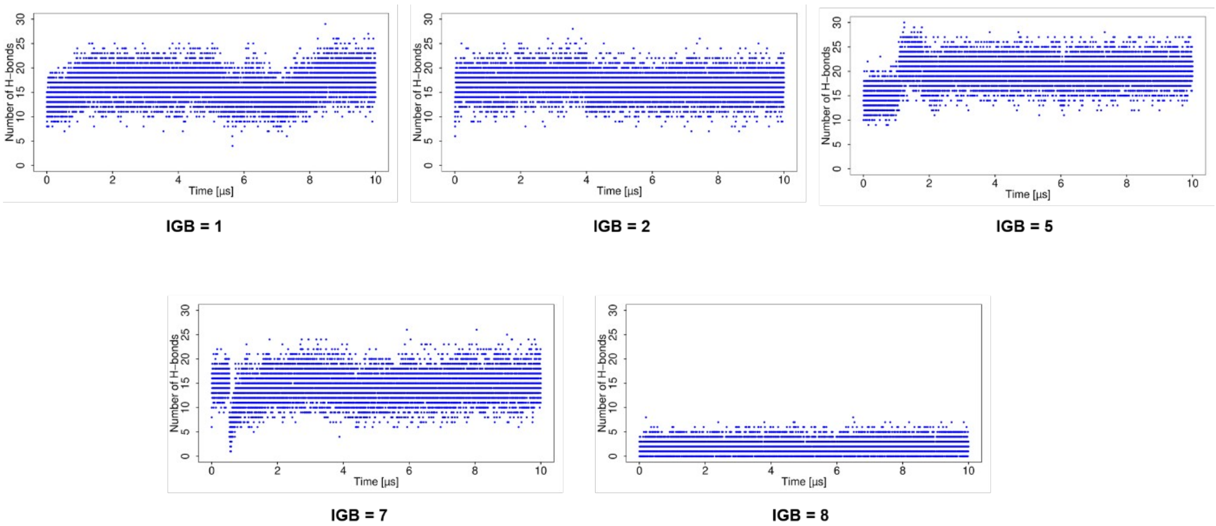

Figure S4. Number of H-bonds obtained for Basic Fibroblast Factor-HP complex in MD simulations using implicit water models.

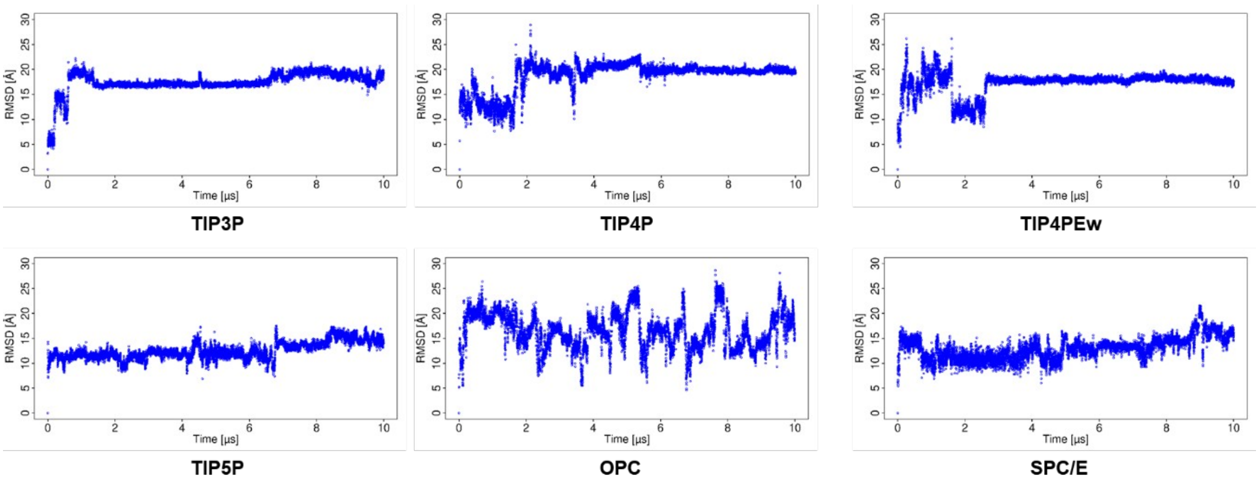

Figure S5. RMSD of the ligand obtained for Cathepsin K-CS complex in MD simulations using explicit water models.

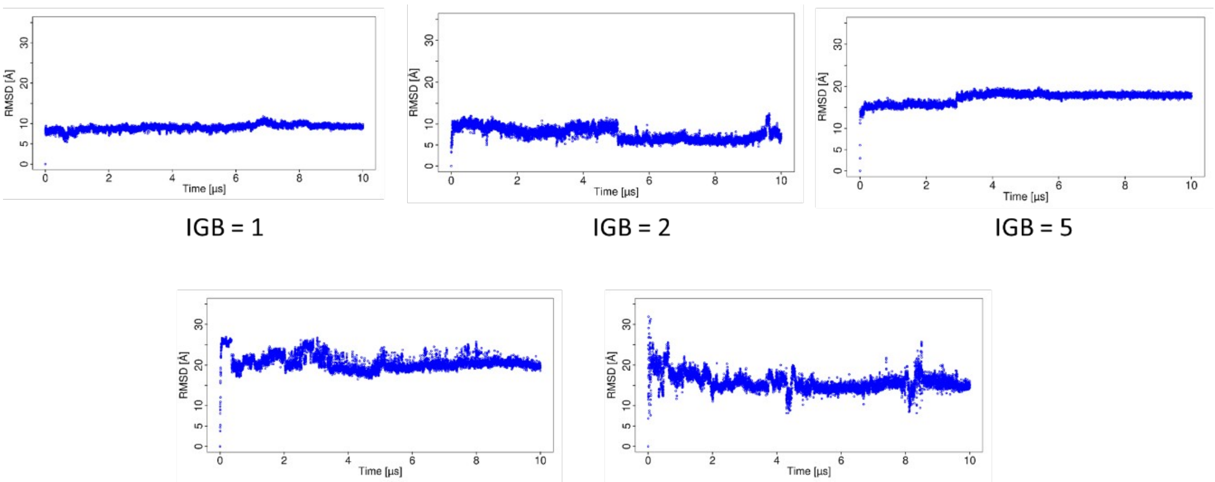

Figure S6. RMSD of the ligand for Cathepsin K-CS complex in MD simulations using implicit water models.

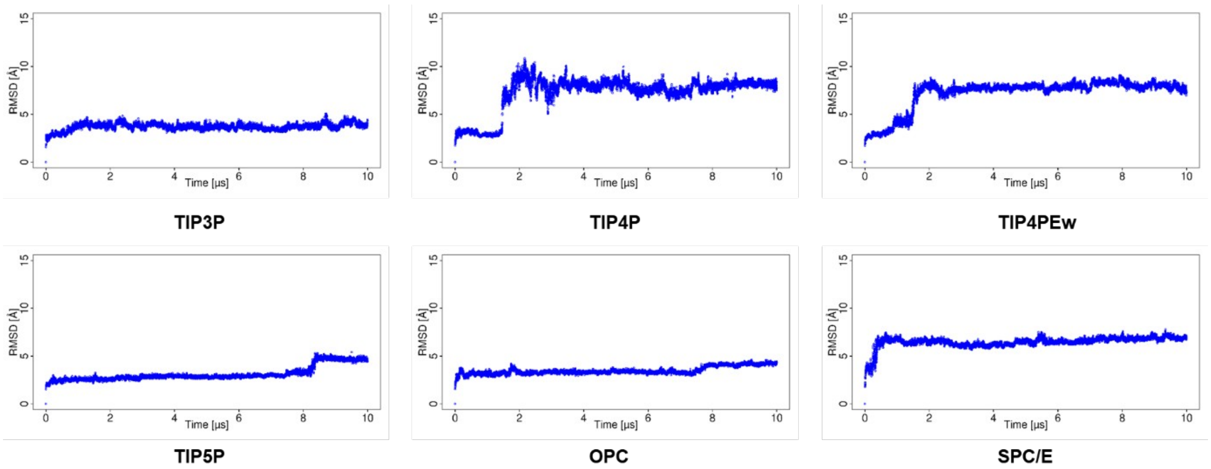

Figure S7. RMSD of the protein obtained for Cathepsin K-CS complex in MD simulations using explicit water models.

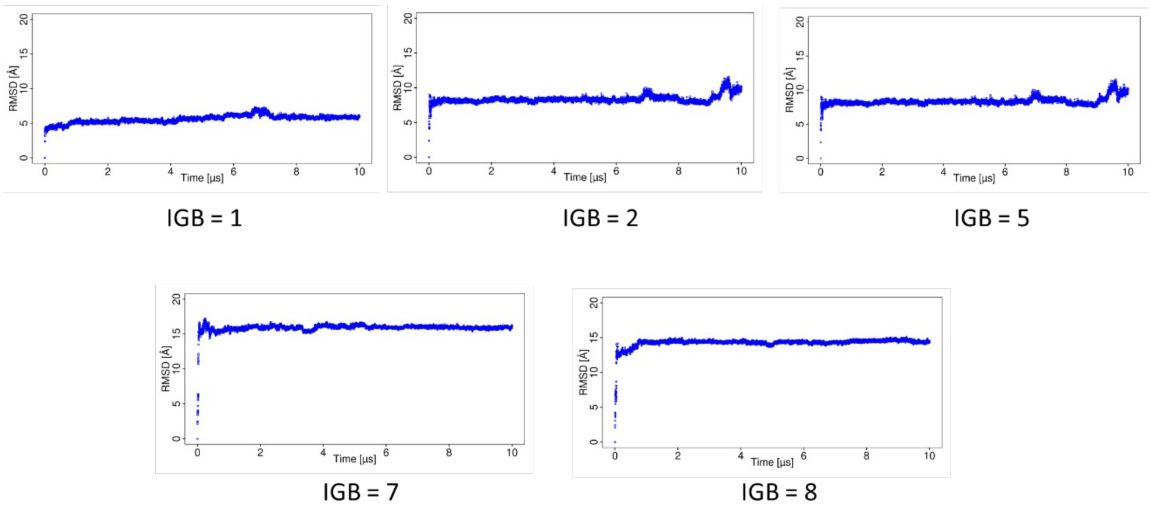

Figure S8. RMSD of the protein obtained for Cathepsin K-CS complex in MD simulations using implicit water models.

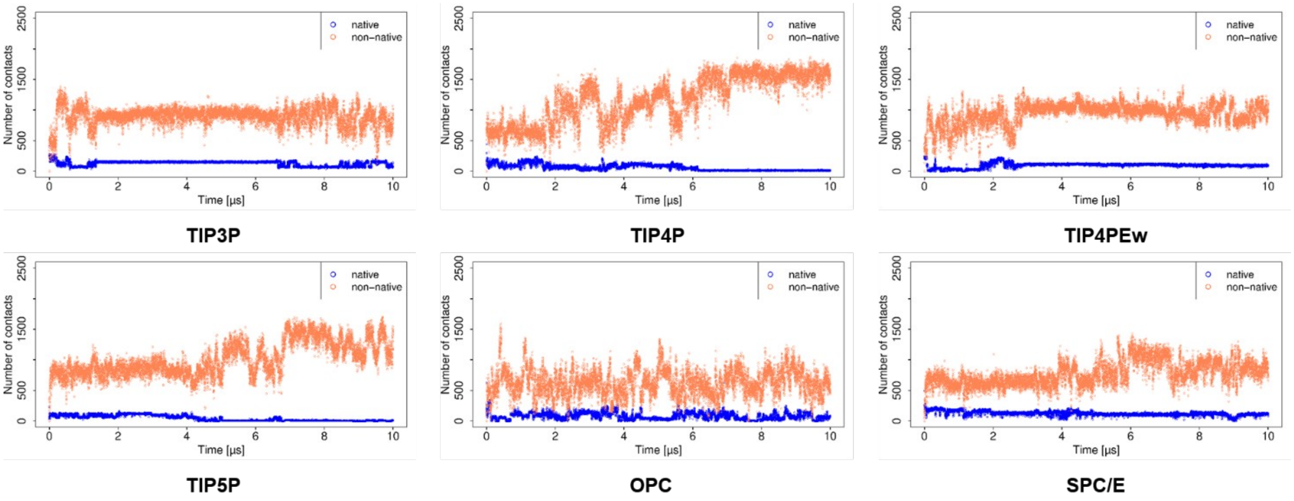

Figure S9. Number of contacts obtained for Cathepsin K-CS complex in MD simulations using explicit water models.

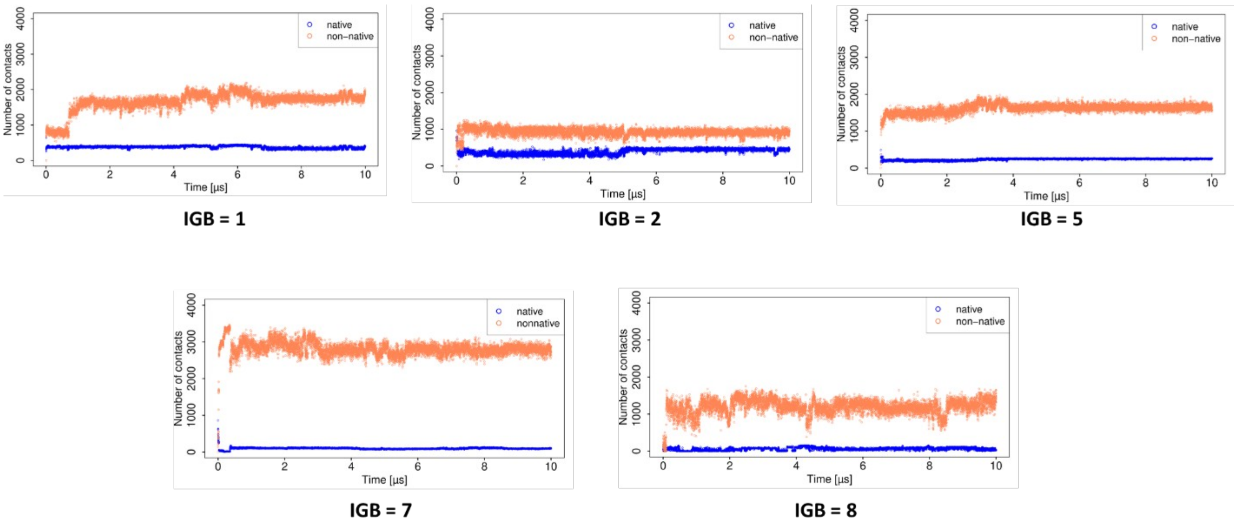

Figure S10. Number of contacts obtained for Cathepsin K-CS in MD simulations using implicit water models.

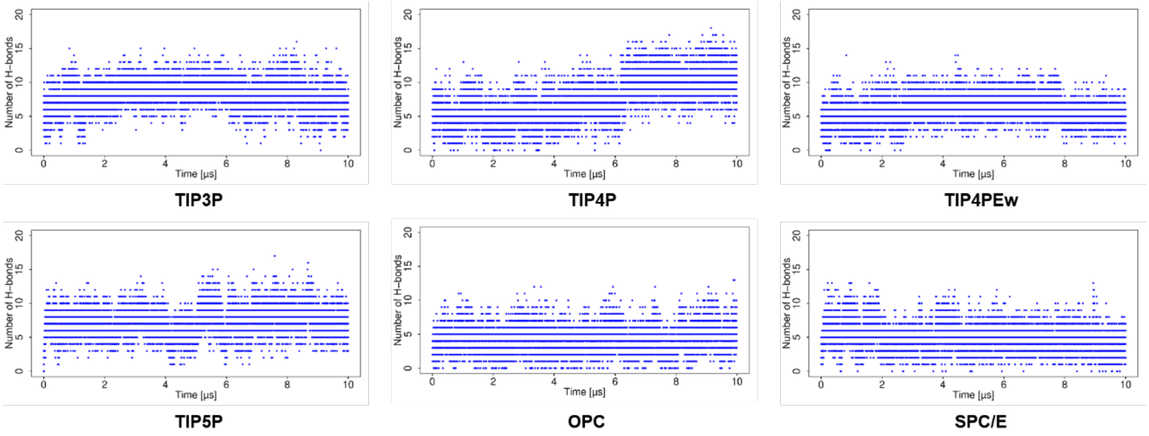

Figure S11. Number of H-bonds obtained for Cathepsin K-CS complex in MD simulations using explicit water models.

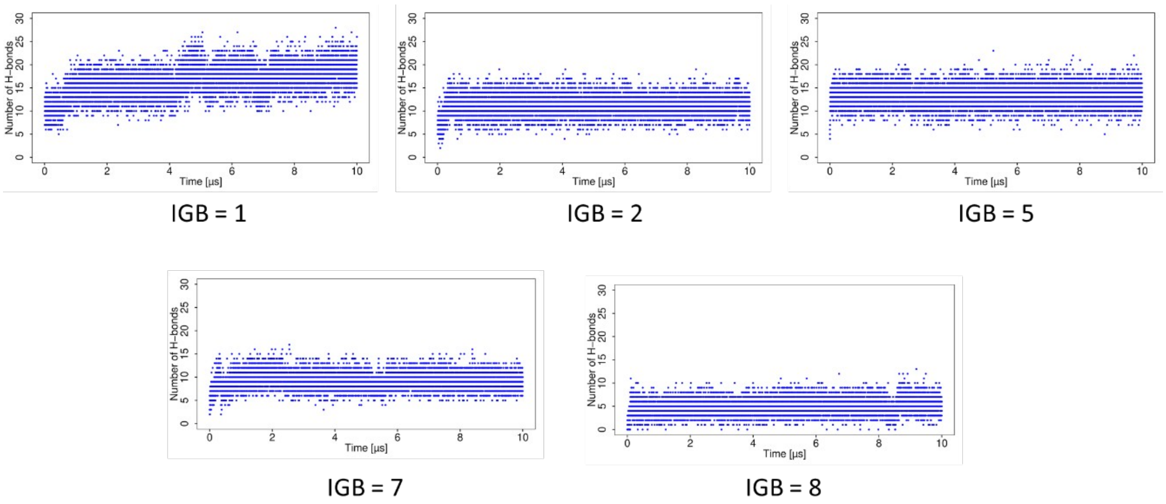

Figure S12. Number of H-bonds obtained for Cathepsin K-CS complex in MD simulations using implicit water models

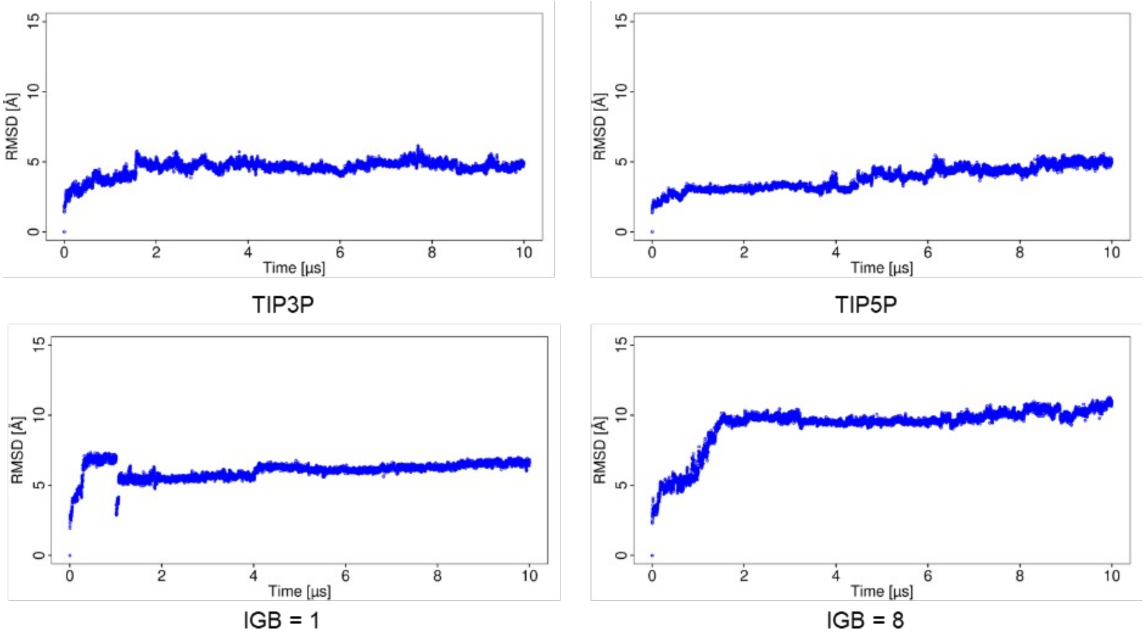

Figure S13. RMSD of the protein obtained for CD44-HA complex.

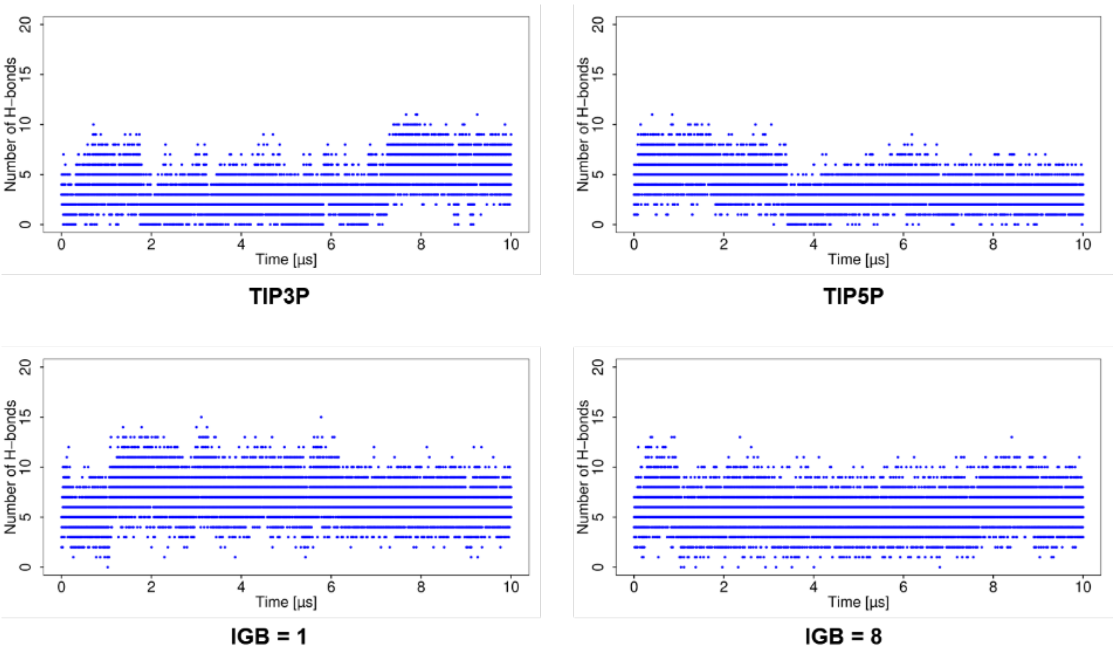

Figure S14. Number of H-bonds obtained for CD44- HA complex.

Table S1. RMSD (mean and standard deviation, Å) of GAG and protein in Basic Fibroblast Factor-HP complex in MD simulations using different water models.

Water model	GAG RMSD	Protein RMSD
TIP3P	7.9 ± 3.3	2.2 ± 0.1
TIP4P	3.4 ± 0.9	2.5 ± 0.3
TIP4PEw	4.0 ± 1.5	2.3 ± 0.2
TIP5P	4.5 ± 1.7	2.3 ± 0.2
OPC	6.4 ± 2.0	2.1 ± 0.2
SPC/E	6.2 ± 1.8	2.1 ± 0.2
IGB=1	4.7±0.6	2.6± 0.4
IGB=2	6.3 ± 0.9	5.8 ± 1.2
IGB=5	3.6 ± 0.4	3.6 ± 0.5
IGB=7	15.6 ± 1.9	10.0 ±0.7
IGB=8	17.9 ± 7.5	6.5±1.1

Table S2. RMSD (mean and standard deviation, Å) of GAG and protein in Cathepsin K-CS complex in MD simulations using different water models.

Water model	GAG RMSD	Protein RMSD
TIP3P	17.5 ± 2.2	3.7 ± 0.4
TIP4P	18.8 ± 2.9	7.2 ± 1.9
TIP4PEw	17.3 ± 2.3	7.2 ± 1.6
TIP5P	12.5 ± 1.7	3.1 ± 0.8
OPC	16.5 ± 3.6	3.5 ± 0.4
SPC/E	12.9 ± 2.2	6.5 ± 0.6
IGB=1	9.1 ± 0.7	5.6 ± 0.5
IGB=2	7.7 ± 1.6	8.4 ± 0.5
IGB=5	17.3 ± 1.2	7.9 ± 0.6
IGB=7	20.6 ± 1.9	15.9 ± 0.6
IGB=8	15.8 ± 2.1	14.3 ± 0.7

Table S3. RMSD (mean and standard deviation, Å) of GAG and protein in CD44-HA complex in MD simulations using different water models.

Water model	GAG RMSD	Protein RMSD
TIP3P	19.3 ± 2.3	4.5 ± 0.6
TIP5P	10.1 ± 5.2	3.8 ± 0.8
IGB=1	17.7 ± 5.6	6.0 ± 0.6
IGB=8	11.5 ± 1.4	9.3 ± 1.6
